# Cytotoxic T lymphocytes require transcription for infiltration but not target cell lysis

**DOI:** 10.15252/embr.202357653

**Published:** 2023-10-20

**Authors:** Arianne C Richard, Claire Y Ma, John C Marioni, Gillian M Griffiths

**Affiliations:** ^1^ Cambridge Institute for Medical Research University of Cambridge Cambridge UK; ^2^ Cancer Research UK Cambridge Institute University of Cambridge Cambridge UK; ^3^ European Molecular Biology Laboratory European Bioinformatics Institute (EMBL‐EBI) Hinxton UK; ^4^ Present address: Immunology Programme The Babraham Institute Cambridge UK

**Keywords:** chemotaxis, cytolysis, cytotoxic T cell, infiltration, transcription, Chromatin, Transcription & Genomics, Immunology, Signal Transduction

## Abstract

Effector cytotoxic T lymphocytes (CTLs) are critical for ridding the body of infected or cancerous cells. CTL T cell receptor (TCR) ligation not only drives the delivery and release of cytolytic granules but also initiates a new wave of transcription. In order to address whether TCR‐induced transcriptomic changes impact the ability of CTLs to kill, we asked which genes are expressed immediately after CTLs encounter targets and how CTL responses change when inhibiting transcription. Our data demonstrate that while expression of cytokines/chemokines and transcriptional machinery depend on transcription, cytotoxic protein expression and cytolytic activity are relatively robust to transcription blockade, with CTLs lysing nearby target cells for several hours after actinomycin D treatment. Monitoring CTL movement among target cells after inhibiting transcription demonstrates an infiltration defect that is not rectified by provision of exogenous cytokine/chemokine gradients, indicating a cell‐intrinsic transcriptional requirement for infiltration. Together, our results reveal differential molecular control of CTL functions, separating recruitment and infiltration from cytolysis.

## Graphical abstract

Cytotoxic T lymphocytes initiate *de novo* transcription immediately after target encounter. While already primed for cytolysis, these transcriptomic changes enable cytokine/chemokine expression and infiltration between target cells, revealing differential molecular controls for cytolysis and recruitment.

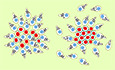

## Introduction

CD8^+^ cytotoxic T lymphocytes (CTLs) form a critical arm of the immune response to intracellular pathogens and cancer. In naïve CD8^+^ T cells, T cell receptor (TCR) recognition of a peptide–MHC (pMHC) ligand complex triggers a complex signalling network that can drive metabolic shifts and transcriptional and translational activation (Ellisen *et al*, 2001; Araki *et al*, 2017; Tan *et al*, 2017; Howden *et al*, 2019; Chapman *et al*, 2020; Mastrogiovanni *et al*, 2020; Jurgens *et al*, 2021). In differentiated effector CTLs, TCR‐induced signalling further initiates cytokine secretion and cytolytic processes to kill the antigen‐presenting cell (Harty *et al*, 2000). Effector CTLs carry pre‐formed cytolytic granules, composed of modified lysosomes containing granzymes and perforin (Dieckmann *et al*, 2016). TCR ligation triggers polarisation of the centrosome and cytolytic granules, resulting in granule secretion within minutes (Ritter *et al*, 2015). Early work demonstrated that secretion of the cytolytic protein GMZA occurred independently of transcription over a 4‐h time‐frame (Fortier *et al*, 1989), while more recent work inhibiting mitochondrial translation demonstrated that new protein synthesis is required for CTLs to sustain killing beyond 4 h (Lisci *et al*, 2021). Whether CTL killing requires *de novo* transcription and how *de novo* transcription during killing feeds into different aspects of the cytolytic process is unclear.

The “near‐instantaneous” nuclear translocation of the transcription factor NFAT upon TCR ligation (Marangoni *et al*, 2013) suggests that there may be a role for *de novo* transcription upon target cell encounter. We therefore examined the impact of target cell recognition on the murine CTL transcriptome immediately after TCR engagement and compared the transcriptional requirements of different TCR‐induced functions. We found that cytolytic protein expression, granule release, and cytolytic activity were remarkably robust to transcriptional blockade. In contrast, transcription was required for TCR‐induced cytokine and chemokine production, as well as infiltration among target cells, making it a critical process for amplifying CTL recruitment and target cell engagement.

## Results and Discussion

### Transcriptomic changes following effector CTL challenge impact transcriptional regulation and cell–cell communication

**Figure 1:**
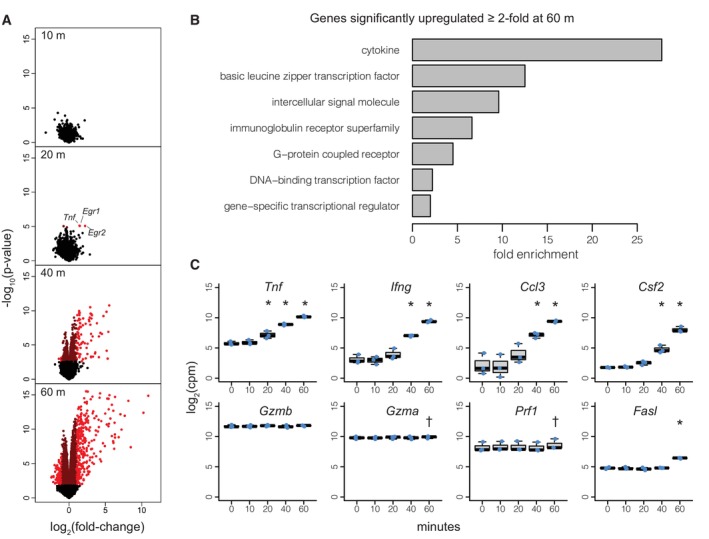
Stimulation of effector CTLs drives upregulation of cytokine mRNAs *In vitro*‐differentiated effector OTI CTLs were stimulated with plate‐bound anti‐CD3 for the indicated times and assayed by RNA‐seq. Figure depicts data from three biological replicates. See Materials and Methods for statistical analysis.
Volcano plots show differential expression at each time point versus resting cells (0 m), plotting the log_2_ fold‐change versus −log_10_ *P*‐value for each gene. Bright and dark red highlight genes that are significantly differentially expressed (FDR < 0.05) with absolute value of the log_2_‐fold change greater or less than 1, respectively.Protein class enrichment of genes from (A) that were significantly differentially expressed (FDR < 0.05) and upregulated at least 2‐fold at 60 min.Gene expression profiles of selected genes are depicted. Each dot represents one of three biological replicates for each time point. Boxplots depict the median and interquartile range, with whiskers extending to the range of the data. * and ^†^ denote conditions in which the gene was significantly differentially expressed (FDR < 0.05) with absolute value of the log_2_‐fold change greater or less than 1, respectively.
Source data are available online for this figure.

To examine transcriptomic changes induced by TCR engagement in effector CTLs, we performed RNA‐sequencing (RNA‐seq) on CTLs stimulated for 0 (unstimulated), 10, 20, 40 and 60 min with anti‐CD3. Differential expression analyses revealed upregulation of *Tnf* and the immediate early genes (IEGs) *Egr1* and *Egr2* as quickly as 20 min after stimulation (Fig 1A, Dataset EV1). Expression of these genes is known to be controlled by activity of pre‐formed transcription factors, specifically NFAT, which rapidly moves to the nucleus following TCR‐induced calcium flux, and ELK1, which is downstream of the MEK/ERK pathways (Goldfeld *et al*, 1993; Shao *et al*, 1997; Falvo *et al*, 2010; Bahrami & Drablos, 2016; Trebak & Kinet, 2019).

**Figure EV1:**
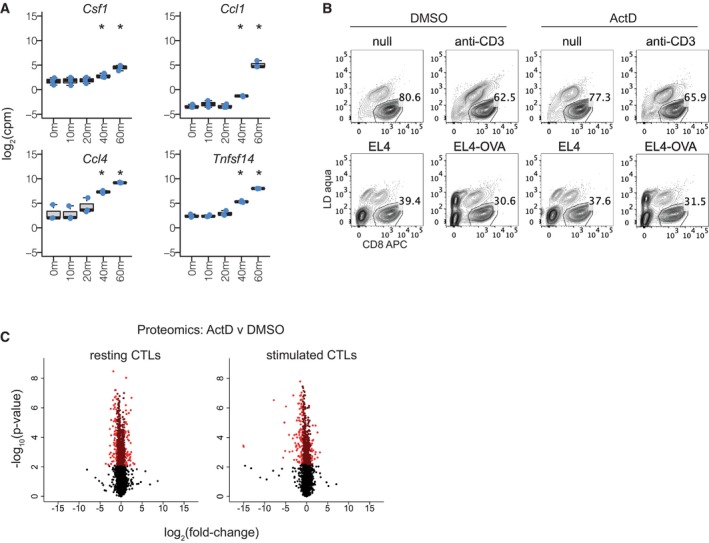
CTL gene expression, and viability and protein changes in the presence of actinomycin D Gene expression profiles of additional selected genes from RNA‐seq analysis depicted in Fig 1. Each dot represents one of three biological replicates for each time point. Boxplots depict the median and interquartile range, with whiskers extending to the range of the data. * denotes conditions in which the gene was significantly differentially expressed (FDR < 0.05) with absolute value of the log_2_‐fold change greater than 1.CTLs were stimulated with or without plate‐bound anti‐CD3 (top), or antigen‐pulsed versus unpulsed EL4 target cells (EL4‐OVA or EL4, respectively, bottom) for 4 h before measuring cell viability by flow cytometry. Cells were additionally treated with actinomycin D (ActD, right) or DMSO vehicle control (left). To test the impact of ActD on CTL viability, data was left completely unfiltered (including debris and target cells where present) and a gate set around living CD8^+^ CTLs using anti‐CD8 antibodies and a live‐dead (LD) stain. Lower percentages with stimulation reflect restimulation‐induced cell death and/or cytolytic death, and lower percentages in the bottom panels reflect the presence of EL4 target cells among CD8^−^ cells. Results are representative of four biological replicates.Volcano plots show results of differential expression analyses from proteomics data in Fig 2, comparing treatment with ActD versus DMSO in resting CTLs (top) and CTLs stimulated for 4 h with anti‐CD3 (bottom). Bright and dark red highlight proteins that are significantly differentially abundant (FDR < 0.05) with absolute value of the log_2_‐fold change greater or less than 1, respectively.

By 60 min after stimulation, extensive transcriptome remodelling had taken place (Fig 1A, Dataset EV1). These changes likely reflect both new transcription and altered transcript degradation (Cheadle *et al*, 2005). Upregulated genes were enriched for cytokines (including chemokines) and those involved in transcriptional regulation (Fig 1B). In contrast to cytokines, expression of genes encoding the cytotoxic proteins granzymes (e.g. *Gzmb*, *Gzma*) and perforin (*Prf1*) exhibited minor or no significant changes in expression during the first hour of stimulation (Figs 1C and EV1A), and expression of the apoptosis‐inducing cytokine *Fasl* was upregulated only after 60 min. Thus, the rapid changes in gene expression after effector CTL challenge primarily impact transcriptional regulation and cell–cell communication, not production of cytolytic granule components.

### Cytokines, including chemokines, are transcriptionally upregulated after CTL challenge

To characterise the role of transcription in TCR‐induced protein expression changes, we used mass spectrometry to profile the proteome of effector CTLs at rest and after 4 h of stimulation with anti‐CD3, in the presence or absence of actinomycin D, which blocks RNA synthesis (Reich *et al*, 1961). At this time point, viability was not substantially affected by actinomycin D treatment (Fig EV1B). In both resting and stimulated CTLs, actinomycin D significantly altered the expression of hundreds of proteins, with more extreme downregulation effects in stimulated cells (Dataset EV2, Fig EV1C).

**Figure 2:**
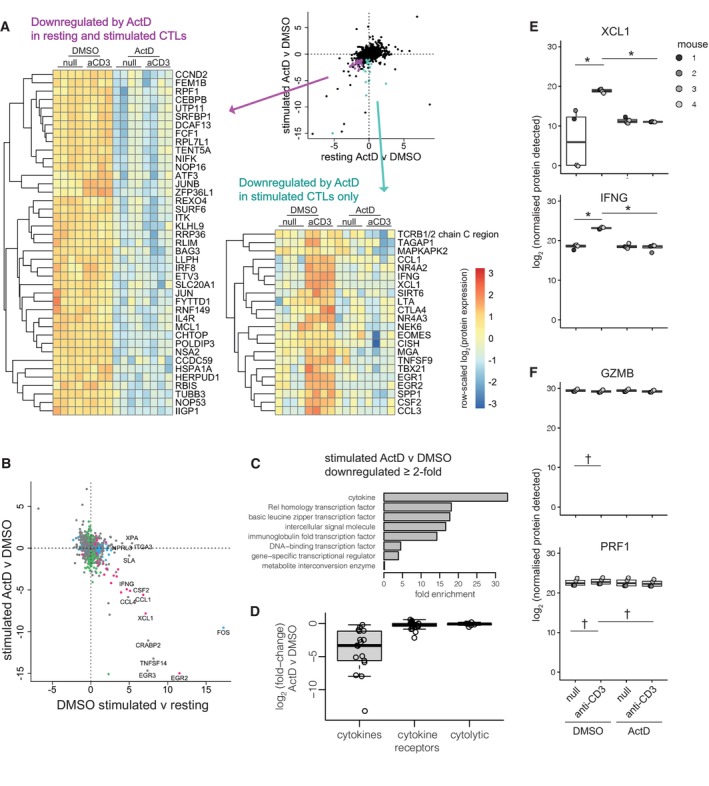
Strong upregulation of cytokine and transcription factor proteins with TCR stimulation is blocked by actinomycin D OTI CTLs were stimulated with or without plate‐bound anti‐CD3, in the presence of actinomycin D (ActD) or DMSO vehicle control, for 4 h before proteome profiling by mass spectrometry. Figure depicts data from four biological replicates. See Materials and Methods for statistical analyses.
AEffect size plot (top right) depicts the log_2_‐fold change for each protein from differential expression analyses of ActD versus DMSO treatment in resting (*x*‐axis) and stimulated (*y*‐axis) CTLs. Proteins strongly downregulated by ActD in both resting and stimulated CTLs (FDR < 0.05, log_2_‐fold change < −1) are highlighted in purple and depicted by heatmap (left). Proteins strongly downregulated by ActD in stimulated CTLs (FDR < 0.05, log_2_‐fold change < −1) but not in resting CTLs (95% confidence interval contains 0) are highlighted in turquoise and depicted by heatmap (bottom right).BEffect size plot depicts the log_2_‐fold change in protein expression in DMSO‐treated stimulated versus resting CTLs on the *x*‐axis, and in stimulated CTLs treated with ActD versus DMSO on the *y*‐axis. Blue dots mark genes significantly differentially expressed in the *x*‐axis comparison, green dots mark genes significantly differentially expressed in the *y*‐axis comparison, pink dots mark genes significantly differentially expressed in both, and grey dots mark all other genes. Proteins with log_2_‐fold change > 4 in stimulated versus resting CTLs are labelled.CProtein class enrichment of proteins significantly differentially expressed (FDR < 0.05) and downregulated at least 2‐fold in stimulated CTLs treated with ActD versus DMSO vehicle control.DProtein expression changes in cytokines, cytokine receptors, and cytotoxic molecules in stimulated CTLs treated with ActD versus DMSO. Each dot represents a protein.E, FExpression of (E) selected cytokines/chemokines and (F) cytolytic components. Each dot represents one of four biological replicates for each condition. Differential expression was tested between resting and stimulated DMSO‐treated cells, and between stimulated cells treated with ActD or DMSO. * and ^†^ denote significant differential expression (FDR < 0.05) with absolute value of the log_2_‐fold change greater or less than 1, respectively, for these comparisons. Data information: Boxplots (D–F) depict the median (central band), interquartile range (IQR, box) and most extreme data points that are ≤ 1.5 × IQR from the box (whiskers). Source data are available online for this figure.

Proteins significantly downregulated by actinomycin D in both resting and stimulated CTLs included the AP‐1 family transcription factors JUN and JUNB; the effector maturation‐associated transcription factor IRF8 (Miyagawa *et al*, 2012); the signalling mediator ITK, which plays a role in CTL effector molecule expression, degranulation and cytolytic function (Kapnick *et al*, 2017); and the RNA‐binding protein ZFP36L1, which itself regulates protein expression and has been shown to restrain effector differentiation in CD8^+^ T cells (Petkau *et al*, 2022) (Fig 2A). Proteins affected by actinomycin D only in the context of TCR stimulation included cytokines (e.g. IFNG, XCL1, CCL3, CCL1, CSF2), IEGs (e.g. NR4A2, NR4A3, EGR1, EGR2), and transcription factors known to drive CTL differentiation and/or functional programmes (TBX21 and EOMES) (Pearce *et al*, 2003; Intlekofer *et al*, 2008) (Fig 2A). These proteins were largely characterised by dramatic upregulation upon stimulation, which was absent when transcription was blocked.

We next examined how transcription blockade affected TCR‐induced changes in protein expression. Comparing changes in protein expression induced by 4 h of stimulation with those driven by actinomycin D treatment during stimulation revealed that proteins that underwent the greatest stimulation‐induced upregulation were most affected by transcription blockade (Fig 2B). This suggests that the most extreme changes in protein expression 4 h after TCR stimulation rely on transcription and not simply translation of pre‐existing mRNAs.

Enrichment analysis of proteins downregulated by actinomycin D treatment in stimulated cells showed a strong impact on expression of cytokines, including chemokines (Fig 2C–E), as has been previously described for IFNG and IL2 (Fortier *et al*, 1989; Salerno *et al*, 2017). In contrast, expression of cytolytic proteins was maintained after 4 h of blocking transcription in both the presence and absence of TCR stimulation (Fig 2D and F). These data indicate that transcription is not required for granule content maintenance over this time frame.

**Figure EV2:**
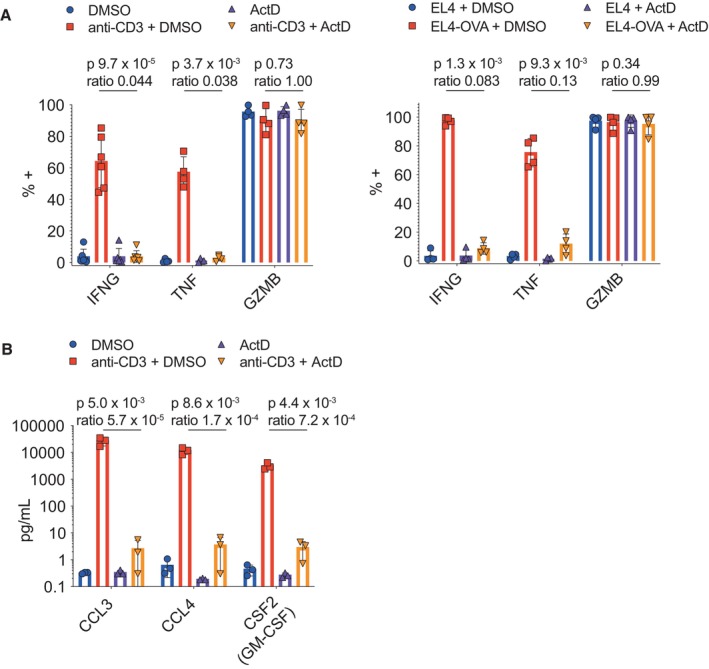
Actinomycin D treatment blocks stimulation‐induced production of cytokines/chemokines at 4 h Flow cytometry and multiplexed cytokine/chemokine bead assays were used to examine the impact of actinomycin D (ActD) treatment on the CTL secretome.
OTI CTLs were stimulated with or without plate‐bound anti‐CD3 (left), or antigen‐pulsed versus unpulsed EL4 target cells (EL4‐OVA or EL4, respectively, right), in the presence of ActD or DMSO vehicle control, for 4 h. IFNG, TNF, and GZMB expression were measured by flow cytometry. For IFNG and TNF staining, secretion was blocked during TCR stimulation to allow intracellular cytokine staining. Bar plots summarise results from six biological replicates per measurement for IFNG under anti‐CD3 stimulation, and four biological replicates for all other measurements.Supernatants were collected from OTI CTLs stimulated with or without plate‐bound anti‐CD3, in the presence of DMSO or ActD, for 4 h, and chemokine secretion was measured by multiplexed bead assay. Plot summarises results from three biological replicates. Data information: (A, B) Bar heights and error bars depict means and standard deviations, respectively. Points show individual biological replicates. *P*‐values by ratio paired *t*‐tests comparing stimulated CTLs treated with ActD or DMSO.

Protein measurements with flow cytometry (IFNG, TNF, GZMB) confirmed that cytokine but not cytolytic protein expression relied on transcription (Fig EV2A). Challenge with antigen‐loaded target cells demonstrated similar effects to anti‐CD3‐mediated challenge (Fig EV2A). Assaying selected cytokines/chemokines (CCL3, CCL4, CSF2) in cell culture supernatants confirmed that their stimulation‐induced secretion required transcription (Fig EV2B).

**Figure EV3:**
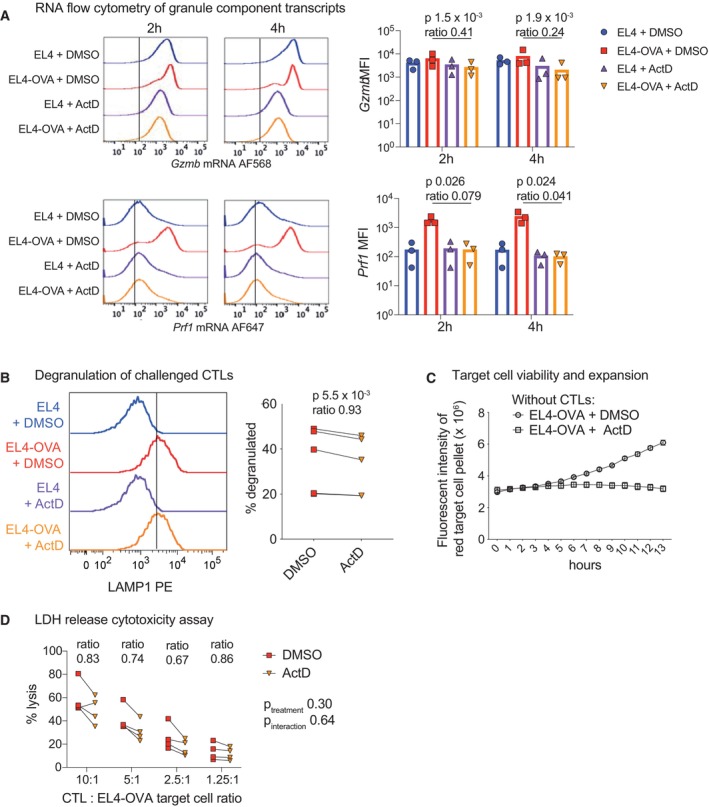
Stimulation‐induced upregulation of *Gzmb* and *Prf1* mRNA is impaired by actinomycin D but degranulation and cytolytic functions are retained for several hours *Gzmb* and *Prf1* mRNA expression was measured by RNA flow cytometry in CTLs challenged at a 1:1 ratio with antigen‐pulsed versus unpulsed EL4 target cells (EL4‐OVA or EL4, respectively), in the presence of actinomycin D (ActD) or DMSO control, for 2 and 4 h. Cell‐based histograms (left) show fluorescence of each marker in one biological replicate, representative of 3. Lines indicate the threshold for positive staining. Bar plots (right) show median fluorescent intensity of mRNA stains among all CTLs in each condition, compiling results from all biological replicates. Bar heights depict means and points show individual biological replicates. *P*‐values by ratio paired *t*‐test comparing stimulated CTLs treated with ActD or DMSO.Degranulation was measured in OTI CTLs challenged as in (A) at a 1:1 CTL:target ratio for 3 h. Histograms (left) depict staining of LAMP1, which is exposed on the CTL surface as cytolytic granules are released, on one biological replicate, representative of 5. Line indicates gate used to delineate degranulated cells. Plot (right) depicts combined data from all biological replicates with values from the same replicate connected by a line (note two replicates are nearly identical and thus not visually separated on plot); *P*‐values by ratio paired *t*‐test.To test the impact of ActD on target cell viability and growth, DMSO or ActD was added to red EL4‐OVA in the absence of any CTLs and red fluorescent intensity monitored by live imaging over time. Points and error bars depict the means and standard deviations of three technical replicates for each measurement. Results are representative of two experimental repeats performed 1 month apart with this cell line.CTLs were challenged as in (A) for 4 h by mixing with target cells at the indicated CTL:target ratios, and target cell lysis measured by release of lactate dehydrogenase (LDH) into the media. Plot depicts results from four biological replicates with lines connecting treatments within each replicate for each ratio. Statistical analysis was performed as a 2‐way ANOVA with Geisser–Greenhouse correction on log‐transformed data to compare ActD/DMSO cytolysis between treatments; *P*‐values are reported for “treatment” and “treatment × CTL:target ratio interaction” terms. The ratio of target cell lysis with ActD versus DMSO treatment is also reported for each CTL:target ratio.

As granzyme and perforin transcripts were abundant in resting CTLs, we asked what happened to these mRNAs when transcription was blocked. Using RNA flow cytometry, we measured *Gzmb* and *Prf1* transcripts in individual CTLs upon target cell challenge in the presence or absence of actinomycin D. We found that CTLs strongly upregulated *Prf1* mRNA at 2 and 4 h after stimulation and that this was blocked by actinomycin D treatment (Fig EV3A). Thus, although the poised cytolytic state persisted at the protein level for several hours in the absence of transcription, stimulation did induce *de novo* expression of genes encoding cytolytic components over time.

Together, our examination of the effects of actinomycin D on CTL protein expression shows that CTLs require transcription to undergo the largest fold‐changes in TCR‐induced protein expression including induction of cytokines/chemokines. However, these cells are fully armed for cytolytic activity by expressing cytotoxic proteins in a poised state for several hours.

### CTL infiltration among target cells is impaired by actinomycin D

**Figure 3:**
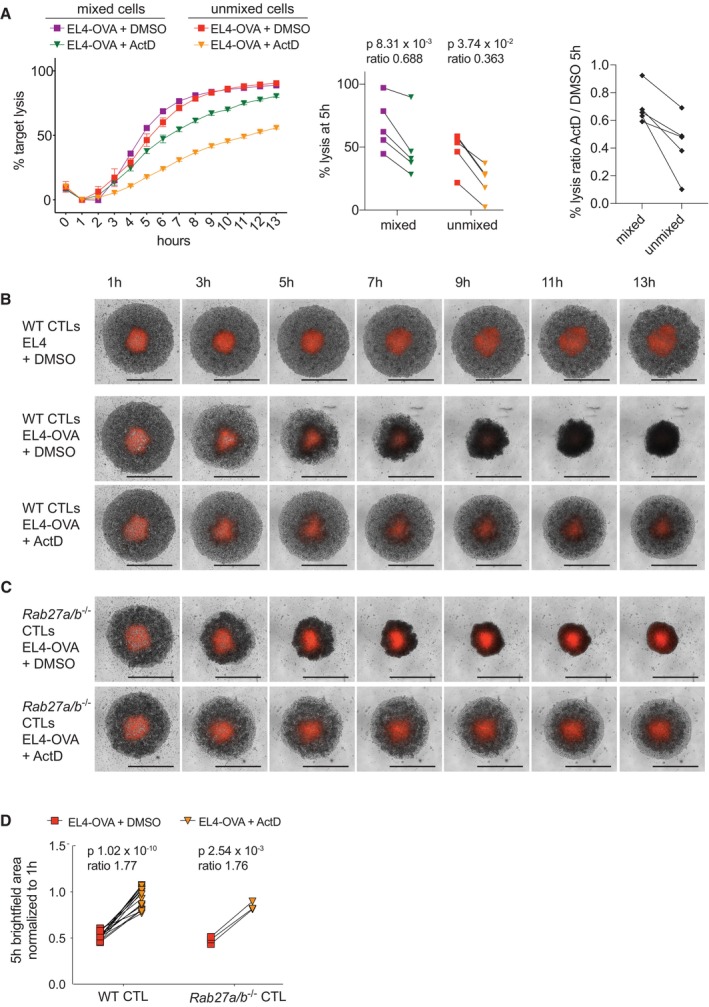
CTLs can kill after actinomycin D treatment but are impaired particularly if required to move into a target cell pellet ACytolytic activity was measured in real time by live cell imaging of OTI CTLs challenged at a 10:1 ratio with antigen‐pulsed versus unpulsed EL4 target cells (EL4‐OVA or EL4, respectively), in the presence of actinomycin D (ActD) or DMSO vehicle control. Target cells expressed a red nuclear protein, allowing quantification of the fraction of target cells remaining. CTLs were either layered on top of the target cells (unmixed) or thoroughly interspersed among target cells (mixed). Plot on the left shows full data from one biological replicate, representative of 5; points and error bars represent the means and standard deviations of three technical replicates for each measurement; error bars not visible when smaller than point size. Plot in the centre depicts combined data from all biological replicates, with cells from the same replicate connected by a line. Plot on the right shows the same data as the ratio of lysis under ActD versus DMSO treatment in mixed and unmixed settings, with cells from the same biological replicate connected by a line.B, C(B) Live imaging was performed as in (A) of CTLs layered on top of red target cells (unmixed). Time‐lapse images are shown of WT OTI CTLs challenged with either unpulsed EL4 target cells (top) or EL4‐OVA (middle) in the presence of DMSO, or EL4‐OVA in the presence of ActD (bottom). Data are representative of 16 biological replicates. Scale bars (black, lower right) = 1 mm. (C) As (B) for RAB27A/B‐deficient OTI CTLs challenged with EL4‐OVA in the presence of DMSO (top) or ActD (bottom). Data are representative of three biological replicates. Original movies (B, C) are provided as Source Data.DThe area of the well occupied by cells in (B, C) was quantified at 5 h and compiled for all biological replicates. Lines connect cells from the same biological replicate under each condition. Data information: *P*‐values (A, D) by ratio paired *t*‐test. Source data are available online for this figure.

Given that CTLs remain poised for cytolytic activity for several hours after blocking transcription, we tested their ability to kill target cells by release of cytolytic granules when transcription was inhibited. Measuring degranulation by LAMP1 antibody uptake, we found no differences with actinomycin D treatment (Fig EV3B). We also asked whether inhibiting transcription impacted CTL killing. Cytolytic activity can depend on both the ability of a CTL to recognise and kill an adjacent target cell and its ability to find a target via migration and infiltration activities. To test both of these capacities, we performed both a “mixed” cytolysis assay, with CTLs interspersed among target cells, and an “unmixed” assay, in which we layered CTLs over a pellet of target cells. We then used live‐imaging to monitor the death of fluorescent target cells over time. Of note, imaging in the absence of CTLs revealed that actinomycin D treatment had minimal intrinsic effect on target cell abundance for the first 5 h after treatment (Fig EV3C). Quantifying target cell death in the presence of CTLs showed that CTLs retained a substantial amount of cytolytic activity in the hours after actinomycin D treatment (Fig 3A), and this was confirmed at different CTL:target cell ratios using a complementary LDH‐release cytotoxicity assay (Fig EV3D). These results were in stark contrast to the near‐ablated cytokine/chemokine production by actinomycin D‐treated CTLs (Figs 2 and EV2). Examining target cell killing at 5 h showed that actinomycin D reduced cytolysis in both mixed and unmixed settings (Fig 3A, left and centre), but the inhibitory effect was consistently greater in the unmixed assay (Fig 3A, right). This indicated that actinomycin D might induce a defect in the ability of CTLs to move and find targets to kill.

**Figure EV4:**
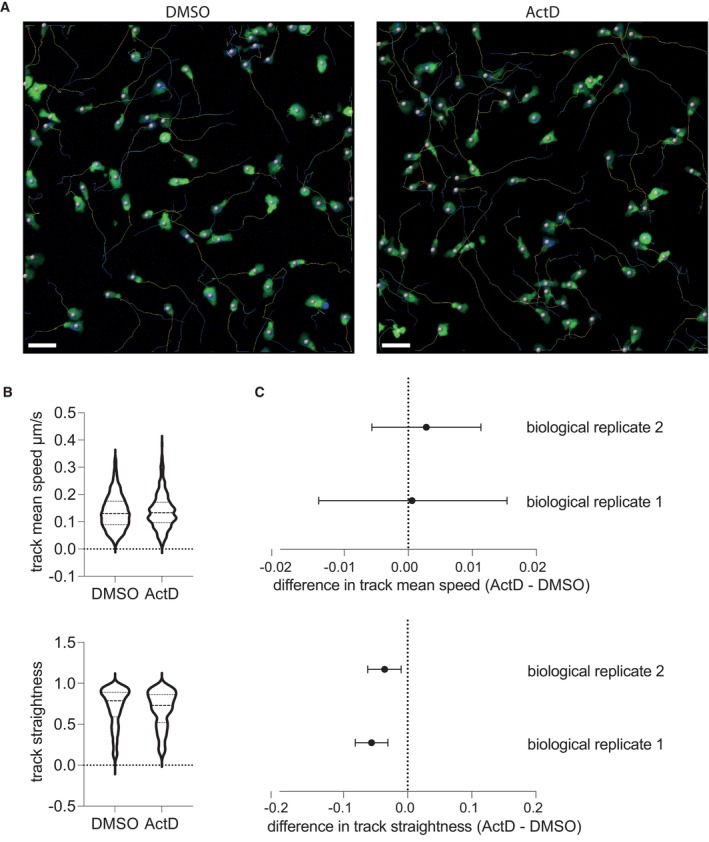
Actinomycin D treatment does not alter basal CTL migration speed CTLs were pre‐treated with actinomycin D (ActD) or DMSO vehicle control and filmed 3–5 h later migrating on ICAM‐1‐coated glass.
Images depict the maximum projection of z‐stacks from the last image of representative movies, with nuclei labelled with spheres and the tracks followed by the migrating CTLs depicted in rainbow; green, CFSE; blue, Hoechst. Representative of 2–5 movies (total 144–422 cells) per condition from each of two biological replicates. Scale bars (white, lower left) = 30 μm.Quantification of track mean speed and track straightness for all cells filmed from one biological replicate.Non‐parametric Hodges‐Lehmann estimate and 95% confidence interval for difference between ActD and DMSO treatment for parameters in (B) in each of the two biological replicates.

We noted that in the unmixed live imaging assay, the area of the well occupied by CTLs contracted as they migrated toward and infiltrated the target cell pellet. However, this contraction was dramatically impeded by actinomycin D treatment (Fig 3B and D), suggesting that transcription might be required for optimal infiltration. In contrast, we found that basal CTL motility on ICAM‐1‐coated coverslips was largely intact after actinomycin D treatment, with no difference observed in the speed of migration and only a small defect in path straightness (Fig EV4, Movies EV1 and EV2), indicating that actinomycin D did not impair random movement.

**Figure EV5:**
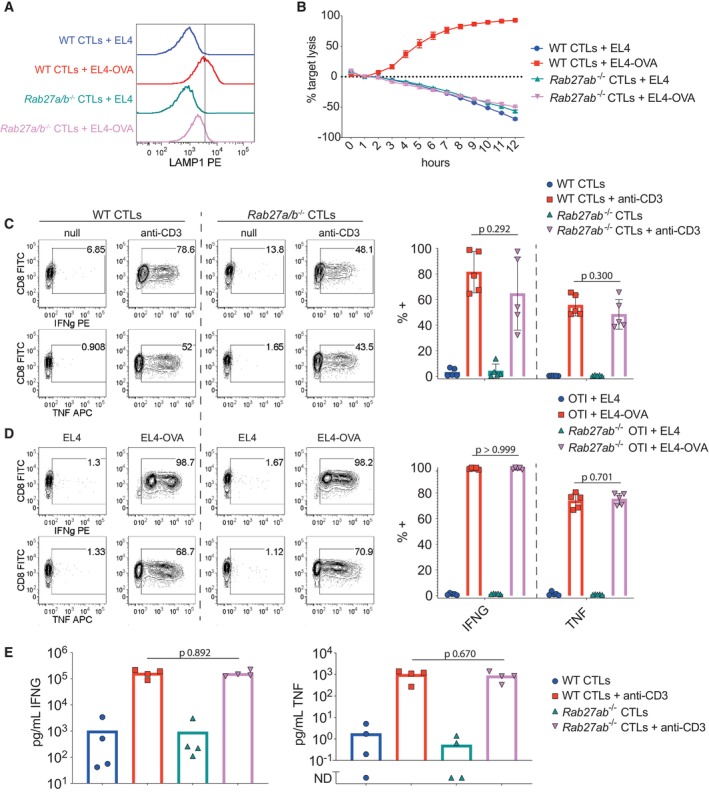
Characterisation of *Rab27a/b* ^−/−^ OTI CTLs ADegranulation was measured in WT OTI and RAB27A/B‐deficient OTI CTLs challenged with antigen‐pulsed versus unpulsed EL4 target cells (EL4‐OVA or EL4, respectively) for 3 h. Histograms depict staining of LAMP1, which is exposed on the CTL surface as cytolytic granules are released. Results are representative of three biological replicates of each genotype.BWT OTI or RAB27A/B‐deficient OTI CTLs were layered on top of EL4‐OVA or EL4 target cells expressing a red fluorescent nuclear protein to measure cytolytic activity through live imaging. Points and error bars depict the means and standard deviations of three technical replicates for each measurement within one biological replicate, representative of 3. Negative death reflects the growth of target cells during the assay.C, DWT OTI and RAB27A/B‐deficient OTI CTLs were challenged (C) with or without plate‐bound anti‐CD3, or (D) with EL4‐OVA or EL4 target cells for 4 h before measuring cytokine expression by flow cytometry. Bar plots (right) depict compiled results from five biological replicates of each genotype. Bar heights and error bars depict means and standard deviations, respectively. Points show individual biological replicates.ESupernatants collected from CTLs challenged as in (C) were assayed for cytokine secretion. Bar plots summarise results from four biological replicates of each genotype. Bar heights depict means and points show individual biological replicates. Data information: *P*‐values (C–E) by Welch's *t*‐test compare stimulated WT with stimulated RAB27A/B‐deficient CTLs.

We next asked whether infiltration was independent of cytolytic activity by using CTLs from OTI *Rab27a* ^ash/ash^ *Rab27b* ^−/−^ mice (referred to as OTI *Rab27a*/*b* ^−/−^), which lack the RAB27A and RAB27B proteins involved in granule exocytosis (Tolmachova *et al*, 2007; Fukuda, 2013). RAB27A/B‐deficient CTLs were incapable of releasing granules or lysing target cells but maintained the ability to secrete cytokines (Fig EV5), as previously shown for RAB27A‐deficient CTLs (Haddad *et al*, 2001; Stinchcombe *et al*, 2001). Live imaging revealed that RAB27A/B‐deficient CTLs infiltrated the target cell pellet in the presence of antigen but, as for wild‐type CTLs, this was blocked by actinomycin D treatment (Fig 3C and D). These results indicate that the transcriptional requirement for infiltration is independent of cytolytic activity and strongly suggest that transcriptional blockade directly impairs infiltration.

### Cell‐intrinsic CTL transcription is required for infiltration

**Figure 4:**
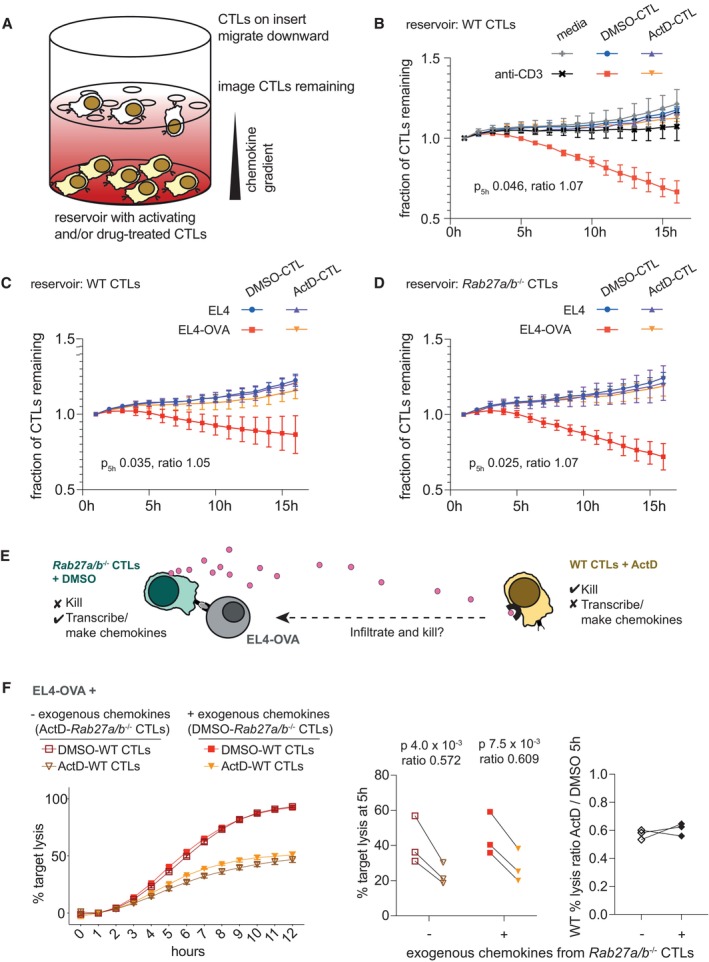
CTLs require transcription to recruit additional CTLs to the site of target recognition and to infiltrate the target cell pellet A–D(A) Cartoon of chemotaxis assay set‐up for panels (B–D.) (B) Chemotaxis assay measured migration of OTI CTLs toward reservoirs containing either media or actinomycin D (ActD)‐ or DMSO‐treated CTLs, with or without plate‐bound anti‐CD3 challenge. Migration is quantified by monitoring the area of the insert occupied by CTLs, which decreases as cells migrate into the reservoir. Plot depicts mean and standard deviation of three biological replicates. (C) Chemotaxis assay as in (B) monitored migration toward reservoirs containing media or actinomycin D (ActD)‐ or DMSO‐treated OTI CTLs challenged with antigen‐pulsed (EL4‐OVA) or unpulsed (EL4) target cells. Plot depicts mean and standard deviation of five biological replicates. (D) As (C) with RAB27A/B‐deficient OTI CTLs in the reservoir. Plot depicts mean and standard deviation of four or five biological replicates per condition. *P*‐values (B–D) compare migration toward reservoirs containing stimulated CTLs treated with ActD versus DMSO at 5 h by ratio paired *t*‐test.ECartoon of co‐culture assay in (F) to test whether cytokines/chemokines secreted by transcriptionally competent RAB27A/B‐deficient CTLs can induce infiltration by transcriptionally inhibited WT CTLs and thereby facilitate target cell killing.FLive‐imaging of cytolytic activity against red EL4‐OVA target cells was performed as in Fig 3 in an unmixed assay. In addition to WT OTI CTLs pre‐treated with ActD or DMSO, we added cytolysis‐incompetent RAB27A/B‐deficient OTI CTLs that were pre‐treated with ActD (null condition) or DMSO (to provide an exogenous cytokine/chemokine gradient). Plot on left depicts full data from one biological replicate, representative of 3; points and error bars represent the means and standard deviations of three technical replicates for each measurement; error bars not visible when smaller than point size. Comparing open to closed shapes shows the impact of having an exogenous cytokine/chemokine gradient from RAB27A/B‐deficient CTLs; comparing triangles to squares shows the impact of ActD on WT CTLs. Original movies are provided as Source Data. Plot in the centre shows combined data from all biological replicates. Lines connect cells from the same biological replicate. *P*‐values by ratio paired *t*‐test. Plot on the right depicts the same data as the ratio of lysis under ActD versus DMSO treatment of WT cells in settings with and without and exogenous chemokine gradient. Lines connect cells from the same biological replicate. Source data are available online for this figure.

During tumour recognition, CTLs undergo a swarming response in which ligand‐stimulated CTLs secrete chemokines, including CCL3 and CCL4, that induce directed migration of other CTLs (Galeano Nino *et al*, 2020). As we had found that secretion of these chemokines required transcription upon target cell encounter (Fig EV2B), we examined how actinomycin D treatment might affect the ability of stimulated CTLs to attract other CTLs. To this end, we pre‐treated CTLs with either actinomycin D or vehicle control and stimulated them with anti‐CD3. We then used these cells as the source of chemotactic signals in a chemotaxis assay and monitored the directed migration of additional, untreated and unstimulated CTLs (Fig 4A and B). Our results showed that treating CTLs with actinomycin D ablated their ability to recruit untreated CTLs (Fig 4B), demonstrating that CTLs must engage in *de novo* transcription to initiate swarming behaviour. Similar results were observed when transcriptionally capable or inhibited CTLs were stimulated with antigen‐loaded target cells instead of anti‐CD3 (Fig 4C).

We next tested RAB27A/B‐deficient CTLs in this chemotaxis assay, as these cells also showed defective infiltration when treated with actinomycin D (Fig 3C). Despite being unable to kill target cells, RAB27A/B‐deficient CTLs successfully recruited other CTLs upon stimulation. However, as with wild‐type CTLs, this effect was dependent on transcription (Fig 4D). Thus, antigen‐induced chemotactic signals occurred independently of cytolytic granule release. Together, our chemotaxis results demonstrate that stimulated CTLs require *de novo* transcription to send soluble signals that attract additional CTLs to the site of target recognition.

Knowing that the production of chemokines was impaired by actinomycin D, we next set up an experiment to test whether provision of an exogenous cytokine/chemokine gradient would improve the killing defect of actinomycin D‐treated CTLs in an unmixed cytolytic activity assay. Our data presented above showed that RAB27A/B‐deficient CTLs could infiltrate the target pellet (Fig 3C and D) and secrete chemokines to induce directed migration (Fig 4D), but not kill target cells (Fig EV5B). Taking advantage of these properties, we used RAB27A/B‐deficient CTLs as a source of cytokines/chemokines and tested whether co‐culture with transcription‐inhibited wild‐type CTLs would improve target cell killing by the latter population when CTLs were layered onto a target cell pellet (Fig 4E). We found that the provision of exogenous chemokines from transcription‐competent RAB27A/B‐deficient CTLs failed to improve target cell killing (Fig 4F). Thus, cytolytic activity depended primarily on whether wild‐type CTLs could transcribe; co‐culture with RAB27A/B‐deficient CTLs made little difference. Together, these data suggest that directed migration/infiltration and subsequent target cell lysis require transcription by the migrating/infiltrating CTL and that provision of exogenous cytokines/chemokines cannot overcome this.

In this study, we examined the role of transcription in CTL effector functions. We found that altered gene expression can be seen within 20 min of TCR engagement, and profound changes to the transcriptome are apparent within an hour, particularly upregulation of transcripts encoding cytokines and transcriptional machinery. These results demonstrate that transcription factor and RNA‐binding protein activity triggered by TCR stimulation transforms the transcriptome of primary effector CTLs in less than an hour. This is consistent with previous observations after PMA and ionomycin stimulation in the Jurkat T cell line (Cheadle *et al*, 2005).

Blocking transcription with actinomycin D, we found that proteins that undergo the most substantial TCR‐induced upregulation at 4 h require transcription (e.g. IFNG, XCL1, CCL1, and EGR2, Fig 2B). These results are reminiscent of work in Th1‐differentiated CD4^+^ T cells, which showed that most genes upregulated upon TCR stimulation rely on recruitment of RNA polymerase II and *de novo* transcription (Davari *et al*, 2017). Enrichment analysis of proteins most affected by blocking transcription in our study revealed particularly strong effects on cytokines. Further experiments using flow cytometry and secretion assays showed that stimulation‐induced protein expression of IFNG, TNF, CCL3, CCL4 and CSF2 is nearly ablated by actinomycin D treatment. Our results are concordant with a previous observation that transcription is required for stimulation‐induced IFNG secretion in a 4 h assay (Fortier *et al*, 1989). In contrast, another study found that translation of pre‐existing mRNAs produces substantial amounts of TNF, and to a lesser extent IFNG, in a 2‐h CTL stimulation assay (Salerno *et al*, 2017). This discrepancy may be due to different methods and/or time points for *in vitro* effector CTL differentiation and/or stimulation. Nevertheless, our data indicate that the massive induction of cytokine/chemokine expression that occurs over 4 h of TCR stimulation requires transcription. These results suggest that, upon TCR ligand recognition, effector CTLs make a substantial biosynthetic investment to achieve sustained cell–cell communication.

In contrast to cytokines/chemokines, we found that expression of cytotoxic molecules is largely maintained over several hours of TCR stimulation in the presence of actinomycin D, demonstrating that CTLs are heavily armed for rapid killing of target cells. This finding is supported by earlier findings that actinomycin D does not affect GZMA secretion in a 4‐h stimulation assay (Fortier *et al*, 1989). Furthermore, the stability of cytolytic granule proteins that we observed in unstimulated cells treated with actinomycin D suggests that their maintenance over several hours does not require *de novo* transcription, consistent with the long half‐life of PRF1 protein (Uellner *et al*, 1997). However, transcript abundance of *Prf1* and, to a lesser extent, *Gzmb* did increase 2–4 h after stimulation in an actinomycin D‐susceptible manner, suggests that re‐arming of granules for long‐term serial killing (Isaaz *et al*, 1995; Halle *et al*, 2016; Lisci *et al*, 2021; Weigelin *et al*, 2021) may ultimately be impacted by transcription.

Testing the impact of blocking transcription on CTL cytolytic function, we found that actinomycin D‐treated CTLs can successfully secrete cytolytic granules and lyse co‐mingled target cells. This indicates that the machinery governing this process can function for at least several hours without transcription. The mild impairment of target cell lysis observed in transcription‐inhibited CTLs may reflect effects on proteins involved in other aspects of cytolytic activity, such as signalling (e.g. ITK) or polarisation (e.g. TUBB3) (Fig 2A, Dataset EV2).

In contrast to their robust cytolytic capacity, we found that transcription‐inhibited CTLs are incapable of recruiting additional CTLs to the site of TCR stimulation. Work describing CCL3‐ and CCL4‐driven CTL swarming suggested that chemokine secretion acts as a positive feedback loop, amplifying the CTL response to TCR stimulation (Galeano Nino *et al*, 2020). Thus, while an individual CTL lyses target cells in a relatively linear manner, chemokine secretion allows for non‐linear recruitment of additional CTLs, dramatically and rapidly enhancing the response.

We also found a cell‐intrinsic transcriptional requirement for CTL infiltration of a target cell pellet. Co‐culture experiments using killing‐defective (RAB27A/B‐deficient) CTLs to provide external soluble signals did not induce target pellet cytolysis by actinomycin D‐treated wild‐type CTLs. These data may reflect a need for transcription to allow CTLs to respond to chemokine signals (e.g. molecules involved in signal integration, directed migration, or movement in the confined space between targets) or a feedback loop whereby the infiltrating cell must itself be able to secrete chemokines. Moreover, experiments using killing‐defective CTLs showed that infiltration is independent of cytolytic capacity. Alongside previous work that demonstrated efficient and even deeper tumouroid infiltration by CTLs that did not find cognate antigen (Galeano Nino *et al*, 2020), these data indicate that the machinery governing CTL infiltration is separate from that controlling cytolysis of antigen‐bearing targets.

Our infiltration assay used a minimal system of CTLs dropped on top of tumour target cells such that a small proportion of CTLs was immediately in contact with targets. The CTLs then had to move into the target pellet to access others. Other work investigating CTL infiltration has utilised a collagen matrix embedded with tumour target cells to form a tumouroid structure on top of which additional collagen embedded with CTLs is polymerised (Galeano Nino *et al*, 2020). This model provides a scaffold for the cells to move within and presents an opportunity for space between the cells. This may be a better mimic of the *in vivo* reality for CTLs, but it does not necessarily recapitulate the cellular adjacency, or even cell–cell junctions, that can be features of solid tumours. It is important to note that all simplified models of infiltration that use only CTLs and tumour cells do not, by definition, recapitulate the full tumour microenvironment, with additional cell types and surrounding healthy tissue. As such, interaction with host cell types, such the recently identified reciprocal production of IFNG by CAR‐T cells and IL12 by host cells to promote anti‐tumour activity (Boulch *et al*, 2021), cannot be assayed. However, the simplistic systems remain beneficial for identifying cell‐intrinsic effects, such as the role of homotypic chemotaxis via CCL3 and CCL4 (Galeano Nino *et al*, 2020) and the transcriptional requirement for movement among tumour target cells that we describe here.

Together, our results highlight distinct biosynthetic requirements for effector CTL functions. Cytolytic machinery is expressed in a long‐lived, poised state, and can be readily engaged upon target cell recognition. In contrast, sustained secretion of cytokines, recruitment of additional CTLs via chemokine gradients, and infiltration among target cells all require transcription after TCR stimulation. This set‐up enables unimpeded, targeted cytolysis, while necessitating more complex biosynthetic pathways for functions that can dramatically increase the number of CTLs responding and the number of targets they encounter. Accumulation and movement of CTLs can facilitate repeated hits to each tumour target to ensure clearance (Weigelin *et al*, 2021). On the other hand, accumulated CTLs in an inflammatory environment can undergo antigen‐independent activation and contribute to immunopathology (Whiteside *et al*, 2018). Requiring *de novo* transcription for recruitment and movement among target cells may protect the host by providing additional opportunities for regulation of more wide‐reaching responses.

## Materials and Methods

### Mice

*Tg(TcraTcrb)1100Mjb Rag1* ^tm1Bal/tm1Bal^ (MGI:3054907 and MGI:2448994 alleles, referred to as OTI) mice were bred on a C57BL/6 background. *Rab27a* ^ash/ash^ *Rab27b* ^tm1.2Seab/tm1.2Seab^ mice (MGI:1856656 and MGI:3706986 alleles, a kind gift from Miguel Seabra; Tolmachova *et al*, 2007) were crossed with OTI mice to achieve the OTI *Rab27a* ^ash/ash^ *Rab27b* ^tm1.2Seab/tm1.2Seab^ *Rag1* ^tm1Bal/tm1Bal^ (referred to as OTI *Rab27a/b* ^−/−^) strain. Mice were housed in University of Cambridge establishments in individually ventilated cages and provided with *ad libitum* normal mouse diet and water, sizzle nest bedding, and enrichment. Breeding and maintenance of transgenic mice was carried out under UK Home Office project licence PP5905963. This research has been regulated under the Animals (Scientific Procedures) Act 1986 Amendment Regulations 2012 following ethical review by the University of Cambridge Animal Welfare and Ethical Review Body (AWERB). ARRIVE reporting guidelines have been followed.

### Cell lines

EL4 cells (RRID CVCL_0255) were maintained in DMEM (Gibco) supplemented with 10% heat inactivated FCS (Labtech) and 100 U/ml penicillin/0.1 mg/ml streptomycin (Sigma). Cells were routinely tested for mycoplasma and were negative through the course of this work. EL4 cells stably expressing mTagBFP2‐Farnesyl‐5 (blue EL4s) (Ritter *et al*, 2015) were used as antigen‐presenting target cells for cytokine expression and degranulation assays. Blue EL4s transduced with the non‐perturbing red nuclear marker NucLight‐Red (Essen Bioscience) were grown under selection with 1 μg/ml puromycin (Gibco) and used as antigen presenting cells (blue+red EL4s) for live imaging cytolytic activity assays.

### CTL culture and stimulation

Splenocytes of both male and female mice 2–6 months old were used to generate *in vitro* CTLs. Cells were cultured in complete media, composed of RPMI 1640 (Sigma or Gibco), 10% heat inactivated FCS, 100 U/ml penicillin/0.1 mg/ml streptomycin, 2 mM L‐glutamine (Sigma), 1 mM sodium pyruvate (Gibco), 50 μM β‐mercaptoethanol (Gibco), and 20 ng/ml (≥ 100 U/ml) murine IL‐2 (Peprotech). Spleens were homogenised through a 70 μm filter and cultured in complete media with 10 nM SIINFEKL peptide (Cambridge Bioscience) for 3 days. Media was then changed and cells expanded daily. All experiments were performed on days 6–8 after initial stimulation.

Cytotoxic T lymphocytes were challenged using antibody‐ or target‐cell‐mediated stimulation. For antibody‐mediated stimulation, flat‐bottom tissue culture plates were coated with 1 μg/ml anti‐CD3e (clone 145‐2C11, BD Biosciences, RRID AB_394590) in PBS at 37°C for 1 h. Plates were then washed in PBS and CTLs added. For target cell challenge, EL4 target cells were pulsed with 1 μM of the ovalbumin peptide SIINFEKL at 37°C for 1 h. Target cells were extensively washed to remove residual peptide and plated with CTLs at the indicated ratio. Where indicated, CTLs were challenged in the presence of 5 μg/ml actinomycin D (Sigma) or DMSO vehicle control. Actinomycin D‐mediated transcriptional inhibition is irreversible when administered at μg/ml concentrations (Yung *et al*, 1990), allowing pre‐treatment and mixing of transcription‐inhibited with uninhibited cells in culture. Where co‐culture of transcription‐inhibited and non‐inhibited cells was desired, cells were pre‐treated with 5 μg/ml actinomycin D or DMSO for 1 h before washing and mixing. The great majority of experiments compared CTLs under different treatments, and thus no randomisation was performed as all biological replicates received all treatments. Samples were not blinded unless specified, but experimentation and analyses were performed by applying the same pipelines across samples, regardless of treatment condition. Assay‐specific exclusion criteria for individual data points are detailed below where used. Sample sizes for genomic measurements were planned to enable detection of consistent, substantial changes in expression that would generate hypotheses for subsequent experiments. Sample sizes for other assays were chosen to ensure reproducibility. In some cases, large numbers of replicates were run as controls when testing additional hypotheses.

### RNA‐sequencing

Two million OTI CTLs were stimulated on an antibody‐coated 12‐well plate for 10, 20, 40, and 60 min. Cells were harvested by carefully removing media and adding 350 μl RLT lysis buffer (Qiagen) supplemented with 1% v/v β‐mercaptoethanol. Resting cells were pelleted by centrifugation and lysed in the same manner. Lysates were passed through a Qiashredder (Qiagen) and frozen. RNA was extracted using the RNEasy Mini Kit and on‐column DNA digestion with RNAse‐free DNAse I (both Qiagen). RNA samples from three biological replicates stimulated in independent time courses were sequenced. RNA quality was verified by Bioanalyzer 2100 (Agilent Technologies), and quantity was determined using a Qubit Fluorometer (v. 3, ThermoFisher Scientific). Sample order was randomised before libraries were prepared for sequencing with the TruSeq Stranded mRNA kit (Illumina) and sequenced using paired‐end 150 base‐pair sequencing on 2 lanes of an Illumina HiSeq4000 at the Cancer Research UK Cambridge Institute by staff blinded to sample treatments.

### RNA‐sequencing analysis

Reads were aligned to the mm10 genome (annotation from Ensembl GRCm38 version 91) with Subread (v 1.5.1) (Liao *et al*, 2013) and exonic reads counted using the featureCounts function (Liao *et al*, 2014) of the Rsubread Bioconductor package (v 1.32.0 in R v 3.5.1) (Liao *et al*, 2019), excluding duplicate reads marked by Picard tools (v 1.93). Samples had between approximately 19.3 and 30.2 million unique mapped exonic reads. Quality of the samples was assessed by the percentage of reads mapping, the number of genes detected per sample, and inter‐sample correlation. All 15 samples were of consistent, good quality and used in analyses. Genes were filtered for those expressed in at least 3 samples (> 0.53 cpm, corresponding to at least 10 counts in the smallest sample).

Differential expression analysis was performed using the edgeR Bioconductor package (v 3.32.1 in R v 4.0.4) (Robinson *et al*, 2010; McCarthy *et al*, 2012; Lun *et al*, 2016). Dispersions were estimated and a quasi‐likelihood negative binomial generalised log‐linear model was fitted to the data including mouse and time as covariates. Differential expression between each stimulated time point and the resting (0 min) cells was assessed by quasi‐likelihood F‐test. The false discovery rate was estimated by the Benjamini‐Hochberg procedure. Protein class enrichment among genes upregulated (FDR < 0.05, log_2_(fold‐change) > 1) against a background of all genes included in the differential expression test was performed using the PANTHER Overrepresentation Test (version 16.0, released 2021‐02‐24) (Mi *et al*, 2021) with a Fisher's Exact Test and FDR < 0.05.

### Proteomics

OTI CTLs were cultured on an antibody‐coated or uncoated 6‐well plate in the presence of DMSO or actinomycin D for 4 h. Three million cells per condition were washed twice in ice‐cold PBS and flash‐frozen. Samples were processed by the Fingerprints proteomics facility (University of Dundee) as described in (Lisci *et al*, 2021). Data were processed, searched, and quantified with Spectronaut (Bruderer *et al*, 2015) (v14) using the directDIA option using analysis settings as in (Reyes *et al*, 2021). The directDIA data were searched against a mouse hybrid database from databases in the July 2019 Uniprot release as described in (Marchingo *et al*, 2020).

### Proteomics analysis

Statistical analyses were performed in R (v 4.0.5). Normalisation was performed by median‐centring log‐transformed values across samples. Proteins with no peptides detected or only a single peptide detected in more than 75% of samples were removed. Because detection thresholds vary by protein, the data contained many non‐random (left censored) 0s. A conservative method was therefore used to replace 0 values which can break linear model assumptions before testing for differential expression: for each protein, a value was sampled from *N*(*x* − 0.05*x*, (0.05*x*)^2^), where *x* is the lowest detected log‐transformed value for that protein.

Differential expression testing was performed by fitting a linear model to log‐transformed, normalised data, with covariates for biological replicate and treatment condition. The false discovery rate was estimated across proteins by the Benjamini‐Hochberg procedure. Protein class enrichment among proteins downregulated by actinomycin D treatment during TCR stimulation (FDR < 0.05, log_2_(fold‐change) < −1) was calculated against a background of all proteins included in the differential expression test using the PANTHER Overrepresentation Test (version 17.0, released 2022‐02‐22) (Mi *et al*, 2021) with a Fisher's Exact Test and FDR < 0.05.

### Flow cytometry

Cytotoxic T lymphocytes were restimulated for 4 h using antibody‐ or target‐cell‐mediated TCR stimulation with blue EL4s. For intracellular cytokine staining of IFNG and TNF, GolgiStop (BD Biosciences) was added at the beginning of the assay to prevent secretion. Restimulated CTLs were then stained with the following two panels of surface and intracellular antibodies. Intracellular staining was performed using the eBioscience Foxp3/Transcription Factor Staining Buffer Set (ThermoFisher Scientific). All staining was performed in the presence of FCR blocking antibodies (clone 93, Biolegend, RRID AB_312801). In panel 1, cells were surface‐stained with Zombie NIR fixable viability kit (Biolegend) and CD8 FITC (clone 53‐6.7, Biolegend, RRID AB_312745); cells were then intracellularly stained with IFNG PE (clone XMG1.2, Biolegend, RRID AB_315402) and TNF APC (clone MP6‐XT22, Biolegend, RRIDAB_315429). In panel 2, cells were surface‐stained with Zombie aqua fixable viability kit (Biolegend) and CD8 APC (clone 53‐6.7, Biolegend, RRIDAB_312751); cells were then intracellularly stained with GZMB FITC (clone GB11, Biolegend, RRID AB_2114575). Fluorescence was measured on a BD LSRFortessa (BD Biosciences) and data analysed in FlowJo v9 or v10 (FlowJo, LLC). Cells were gated on forward and side scatter, single cells, live cells, and CD8^+^blue^−^ CTLs (Appendix Fig S1A). Positive gates for cytokine and GZMB expression were based on isotype control staining.

### RNA flow cytometry

Cytotoxic T lymphocyte were restimulated for 2 or 4 h with antigen‐pulsed or unpulsed blue EL4 target cells. Cells were first stained with Zombie NIR fixable viability kit (Biolegend). *Gzmb* and *Prf1* transcripts were then stained using PrimeFlow RNA Assays (VB1‐3031201‐PF and VB10‐3282422‐PF, respectively) and Kit (all ThermoFisher) according to the manufacturer's instructions with elongated incubations to improve signal. Fluorescence was measured on a BD LSRFortessa (BD Biosciences) and data analysed in FlowJo v10 (FlowJo, LLC). Cells were gated on forward and side scatter, single cells, live cells, and blue^−^ CTLs (Appendix Fig S1B). Gates for *Gzmb* and *Prf1* were based on fluorescence‐minus‐one staining.

### Soluble chemokine and cytokine measurements

Secretion was measured in the supernatants of CTLs challenged with antibody‐mediated TCR stimulation for 4 h, in the presence of actinomycin D or DMSO vehicle control where indicated. CCL3, CCL4, GM‐CSF and M‐CSF concentrations were measured by LEGENDplex (Biolegend) assay, according to the manufacturer's instructions and analysed using LEGENDplex software. For samples stimulated with anti‐CD3 in the presence of DMSO only, concentrations were at or above the detection limit of the assay for CCL3, CCL4 and GM‐CSF. To make accurate measurements, supernatant samples were diluted 20‐fold before assaying these analytes. Data were collected on a BD LSRFortessa (BD Biosciences) and analysed using LEGENDplex software (v 7.1, Biolegend). M‐CSF expression was often below the limit of detection and not upregulated with stimulation and so is not shown.

TNF and IFNG concentrations were measured using ELISA MAX Deluxe kits for mouse IFN‐γ and TNF‐α (Biolegend). For all TNF‐α measurements, supernatants were diluted 1:4. For IFN‐γ measurements, supernatants were diluted 1:4 for resting conditions or 1:400 for anti‐CD3‐restimulated conditions to achieve absorbances within the range of the standard curve. Absorbances were read on a Tecan Spark plate‐reader and signal was calculated as absorbance at 570 nm subtracted from that at 450 nm. Cytokine concentrations were interpolated by fitting a cubic polynomial to the standard curve in Prism (v 8.2.0, GraphPad).

### Degranulation assay

Cytotoxic T lymphocytes were mixed with antigen‐pulsed or unpulsed EL4 target cells and incubated for 3 h in the presence of 2 μg/ml anti‐LAMP1 PE (clone eBio1D4B, eBioscience, RRID AB_657555). Cells were moved to ice and stained with FCR blocking antibodies (clone 93, Biolegend), Zombie NIR fixable viability kit (Biolegend), and CD8 FITC (clone 53‐6.7, Biolegend, RRID AB_312745). Fluorescence was measured on a BD LSRFortessa (BD Biosciences) and data analysed in FlowJo v9 (FlowJo, LLC). Cells were gated on forward and side scatter, single cells, live cells, and CD8^+^ CTLs before comparing uptake of anti‐LAMP1 stain (Appendix Fig S1C).

### Live imaging cytolytic activity assay

Cytotoxic T lymphocytes were added 10:1 to antigen‐pulsed or unpulsed blue+red EL4s in 96‐well round‐bottom plates (Corning Ultra‐Low Attachment, or BRAND inertGrade). Cells were treated with actinomycin D or DMSO vehicle control where indicated. All assays were run with three technical replicates. Cells were cultured in an IncuCyte S3 Live Cell Analysis System (Sartorius) that contains a Basler Ace 1920‐155 μm compact camera, imaging brightfield and red (excitation: 655 nm; emission: 681 nm) channels every hour with the 4× objective (resolution 2.82 μm/pixel). Data were analysed using IncuCyte S3 Software v2018‐2021 (Sartorius) spheroid analysis, quantifying the total red integrated intensity from each well as a proxy for the number of live EL4 target cells. Within each well, this intensity was then normalised to the intensity at 1 h (due to substantial settling over the first hour of culture) to quantify the fraction of EL4 cells remaining at each time point and the corresponding percentage death. In one experiment, intensity was normalised to that at 2 h due to a failed 1‐h reading. Wells with bubbles, scratches or debris interfering with fluorescent cell quantification were excluded.

This assay was used to assess cytolytic activity when CTLs and targets were mixed together or when the CTLs were required to infiltrate a pre‐formed target cell pellet. For mixed assays, all cells were added to the plate, mixed together, and centrifuged at 233 × *g* for 1 min to generate a loose pellet of cells. For unmixed assays, EL4 target cells were added to the plate and pelleted at 233 × *g* for 1 min to create a loose pellet before addition of CTLs and a repeat of centrifugation. Due to the U‐bottom shape of the plate, addition of 10× CTLs on top of a 1× pellet of red target cells creates a brightfield halo around the red pellet. To monitor migration toward and infiltration among the target cells, we measured reduction of this area. Importantly, this area reduced with antigen‐pulsed target cells even in the absence of cytolytic activity with RAB27AB‐deficient CTLs, indicating that it was not due to cell death. Brightfield area quantification was always performed using Corning Ultra‐Low Attachment plates for consistency of plate curvature. The area of the largest brightfield object detected at 5 h was normalised to the value at 1 h.

We also used this live imaging system to perform a co‐culture experiment in which we tested whether transcription‐capable RAB27A/B‐deficient CTLs could induce infiltration and target cell killing by transcription‐inhibited wild‐type CTLs. To achieve this, we pre‐treated OTI CTLs and RAB27AB‐deficient OTI CTLs with actinomycin D or DMSO control, washed the cells, and layered the indicated combinations of CTLs in equivalent numbers onto antigen‐pulsed or unpulsed EL4 target cell pellets for a final ratio of 10:10:1, CTL:CTL:EL4.

### LDH release cytotoxicity assay

Cytotoxic T lymphocyte cytolytic activity was measured using the CytoTox 96 Non‐Radioactive Cytotoxicity Assay. CTLs and antigen‐pulsed or unpulsed EL4 target cells were combined at the indicated ratios in RPMI 1640 medium without phenol red (Gibco) supplemented with 2% FCS and penicillin/streptomycin. Cells were incubated for 4 h before measuring LDH (lactate dehydrogenase) release according to the manufacturer's instructions. Colorimetric assay results were read on a SpectraMax M5 using Softmax Pro v.7.1 software. Technical triplicate wells were averaged for each condition and percentage target cell lysis calculated as the difference in death between wells with antigen‐loaded versus unloaded target cells, divided by the maximum death observed in target cells treated with lysis buffer.

### Confocal live imaging

For motility experiments, OTI CTLs were labelled with 5 μM CFSE cell trace marker and treated with actinomycin D or DMSO for 3–5 h before incubating with Hoechst 33342 (ThermoFisher, catalogue no. H3570) nuclear label. Cells were then plated in glass‐bottom imaging petri dishes (35 mm, No. 1.5 coverslip, MatTek,) that had been coated with 0.5 μg/ml ICAM‐1/Fc (R&D Systems). After settling, cells were washed with RPMI 1640 without phenol red (Gibco) supplemented with 10% FCS, 25 mM HEPES (Gibco), 100 U/ml penicillin/0.1 mg/ml streptomycin, and 2 mM L‐glutamine. Dishes were maintained at 37°C and 5% CO_2_ in a stage‐top chamber (Okolab) during imaging. Live imaging was performed on a confocal microscope system (Andor‐Revolution XD) with spinning‐disk unit (CSU‐X1; Yokogawa) with 405 and 488 nm excitation lasers with Borealis Enhanced illumination. Images were captured using a 20× objective (0.75 NA, Leica), and EMCCD iXon Ultra 888 camera with Fusion software (Andor). Z‐stack images (7 slices with 2 μm interval) were acquired every 20 s for a total of 30 images. CTL motility (track speed and track straightness) was quantified with Imaris software (v9.3.1), using spot analysis to track nuclei over time. A small number of obviously dead cells (e.g. a bright, round, unmoving nucleus with negligible cytoplasm) or those appearing in the imaging frame for less than 1 min were excluded from track analysis.

### Chemotaxis

Chemotaxis assays were performed on the IncuCyte S3 Live Cell Analysis System (Sartorius) using the Incucyte Clearview 96‐well Plate for Chemotaxis (Sartorius). The insert plate was coated with 0.5 μg/ml ICAM1‐Fc (R&D Systems). CTLs were plated 5,000 cells per well of the Clearview chemotaxis plate insert. To examine chemotaxis toward CTLs actively killing target cells, a CTL challenge assay was set up as described above in the Clearview chemotaxis plate reservoir. For antibody‐mediated challenge, reservoir wells were coated with anti‐CD3 and CTLs challenged for 1–1.5 h before adding the insert. For target cell‐mediated challenge, CTLs were combined with antigen‐pulsed or unpulsed EL4 target cells at a 10:1 ratio for 1–1.5 h before transfer into the reservoir wells of the chemotaxis plate and adding the insert. To test the impact of blocking transcription on the generation of a chemotactic gradient, reservoir CTLs were pretreated with DMSO or ActD for 35 min and washed thoroughly before use. All conditions were run in at least triplicate. Wells with bubbles or condensation interfering with cell quantification were excluded. Brightfield imaging of the top of the insert was performed every 30 or 60 min using the 10× objective (resolution 1.24 μm/pixel). Data were analysed using IncuCyte S3 Software v2018‐2021 (Sartorius) with the Chemotaxis module to quantify the area of the top of the insert occupied by cells at each time point. Within each well, areas were normalised to that measured at 1 h to control for well‐to‐well variability.

### Additional statistical analysis

Statistical analyses of flow cytometry, secretion, cytolytic activity, and infiltration assays were performed using GraphPad Prism software (v 9). For assays comparing CTLs treated with ActD versus DMSO, two‐sided ratio paired *t*‐tests were used to test deviation of the ratio of ActD versus DMSO measurements within each biological replicate. This allowed for differences in baseline responses between biological replicates while looking for common multiplicative effect sizes. All tests passed a Shapiro–Wilk test for normality, and those with sufficient numbers of samples also passed a Kolmogorov–Smirnov test for normality. Cytokine and chemokine expression in WT versus Rab27A/B‐deficient CTLs were compared by two‐sided Welch's *t*‐tests.

## Author contributions

**Gillian M Griffiths:** Conceptualization; supervision; funding acquisition; writing – original draft; writing – review and editing. **Arianne C Richard:** Conceptualization; formal analysis; investigation; writing – original draft; writing – review and editing. **Claire Y Ma:** Investigation. **John C Marioni:** Software; funding acquisition; methodology; writing – review and editing.

## Disclosure and competing interests statement

JCM has been an employee of Genentech since September 2022.

## Supporting information



Appendix

Expanded View Figures PDF

Movie EV1

Movie EV2

Dataset EV1

Dataset EV2

PDF+

Source Data for Figure 1

Source Data for Figure 2

Source Data for Figure 3

Source Data for Figure 4

## Data Availability

(i) RNA‐seq data: ArrayExpress E‐MTAB‐12083 (https://www.ebi.ac.uk/biostudies/arrayexpress/studies/E‐MTAB‐12083); (ii) Mass spectrometry proteomics data: ProteomeXchange Consortium via the PRIDE (Perez‐Riverol *et al*, 2022) partner repository PXD034920 (https://www.ebi.ac.uk/pride/archive/projects/PXD034920); (iii) Analysis code: GitHub (https://github.com/MarioniLab/CTLstimulation).
