# Supplementary material for: Cytotoxic T lymphocytes require transcription for infiltration but not target cell lysis
Source: EMBO Rep. 2023 Oct 20;24(11):e57653. doi: 10.15252/embr.202357653 (PMC10626425; doi:10.15252/embr.202357653)
Supplement: Supplementary file 1 — Appendix [file EMBR-24-e57653-s010.pdf]

Appendix

Cytotoxic T lymphocytes require transcription for infiltration but not target cell lysis

Arianne C. Richard, Claire Y. Ma, John C. Marioni*, Gillian M. Griffiths*

Table of Contents:

2 – Appendix Figure S1

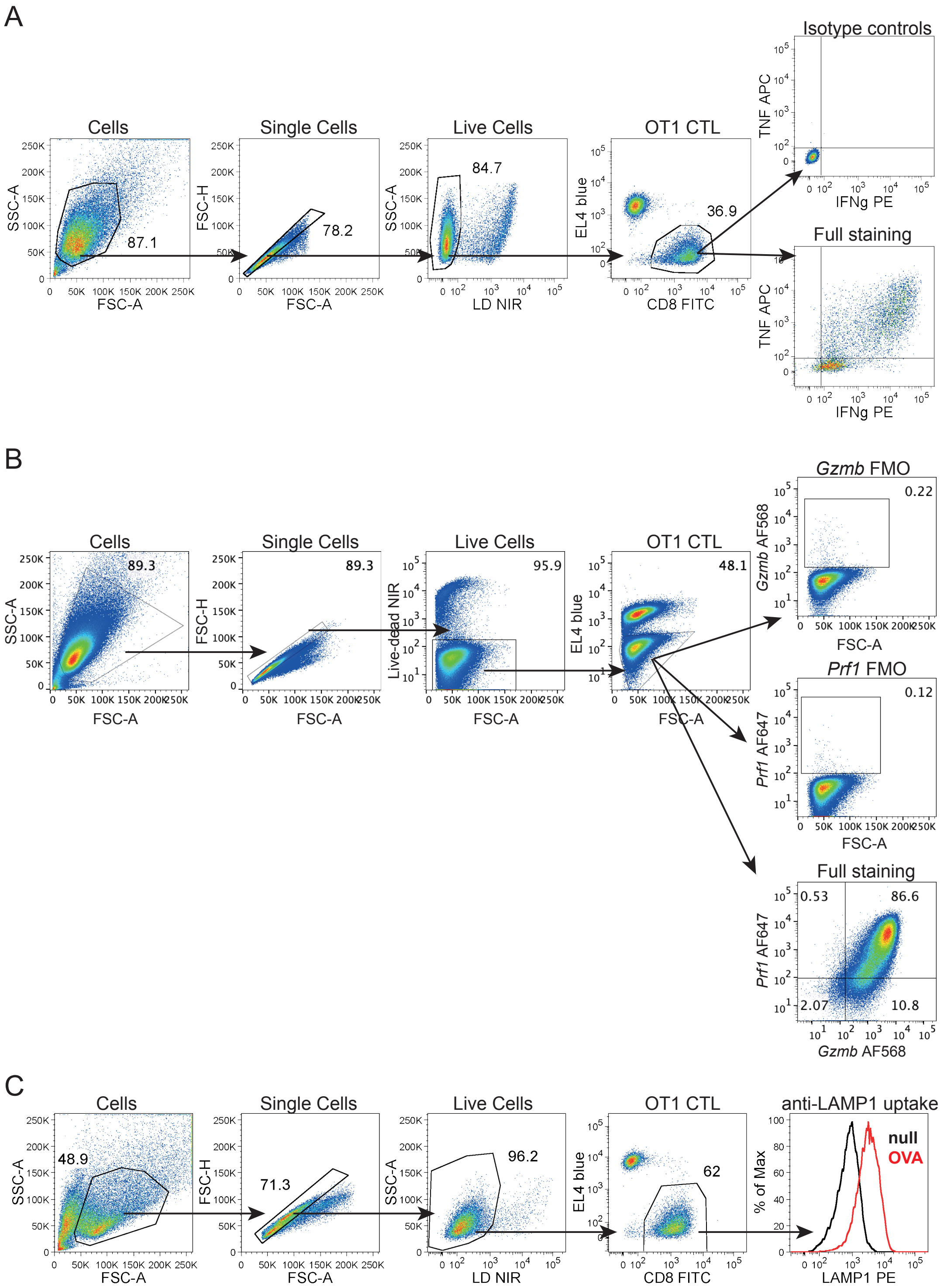

Appendix Figure S1: Flow cytometry gating. Gating for A) cytokine flow cytometry B) RNA flow cytometry and C) degranulation assays.
