# Supplementary material for: Cytotoxic T lymphocytes require transcription for infiltration but not target cell lysis
Source: EMBO Rep. 2023 Oct 20;24(11):e57653. doi: 10.15252/embr.202357653 (PMC10626425; doi:10.15252/embr.202357653)
Supplement: Supplementary file 2 — Expanded View Figures PDF [file EMBR-24-e57653-s005.pdf]

Expanded View Figures

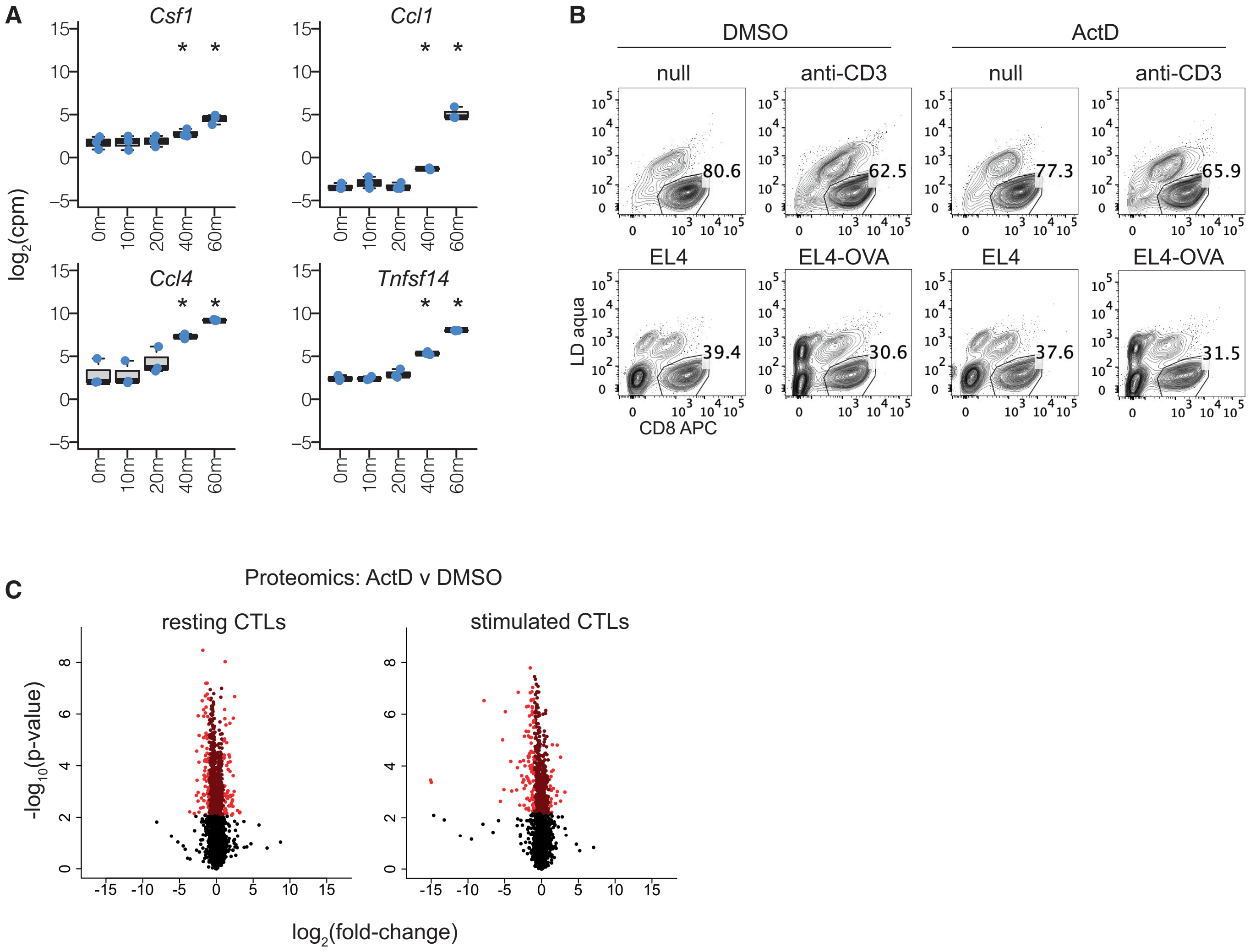

Figure EV1. CTL gene expression, and viability and protein changes in the presence of actinomycin D.

A Gene expression profiles of additional selected genes from RNA-seq analysis depicted in Fig 1. Each dot represents one of three biological replicates for each time point. Boxplots depict the median and interquartile range, with whiskers extending to the range of the data. * denotes conditions in which the gene was significantly differentially expressed (FDR < 0.05) with absolute value of the $\log_2$-fold change greater than 1.

B CTLs were stimulated with or without plate-bound anti-CD3 (top), or antigen-pulsed versus unpulsed EL4 target cells (EL4-OVA or EL4, respectively, bottom) for 4 h before measuring cell viability by flow cytometry. Cells were additionally treated with actinomycin D (ActD, right) or DMSO vehicle control (left). To test the impact of ActD on CTL viability, data was left completely unfiltered (including debris and target cells where present) and a gate set around living $CD8^+$ CTLs using anti-CD8 antibodies and a live-dead (LD) stain. Lower percentages with stimulation reflect restimulation-induced cell death and/or cytolytic death, and lower percentages in the bottom panels reflect the presence of EL4 target cells among $CD8^-$ cells. Results are representative of four biological replicates.

C Volcano plots show results of differential expression analyses from proteomics data in Fig 2, comparing treatment with ActD versus DMSO in resting CTLs (top) and CTLs stimulated for 4 h with anti-CD3 (bottom). Bright and dark red highlight proteins that are significantly differentially abundant (FDR < 0.05) with absolute value of the $\log_2$-fold change greater or less than 1, respectively.

© 2023 The Authors

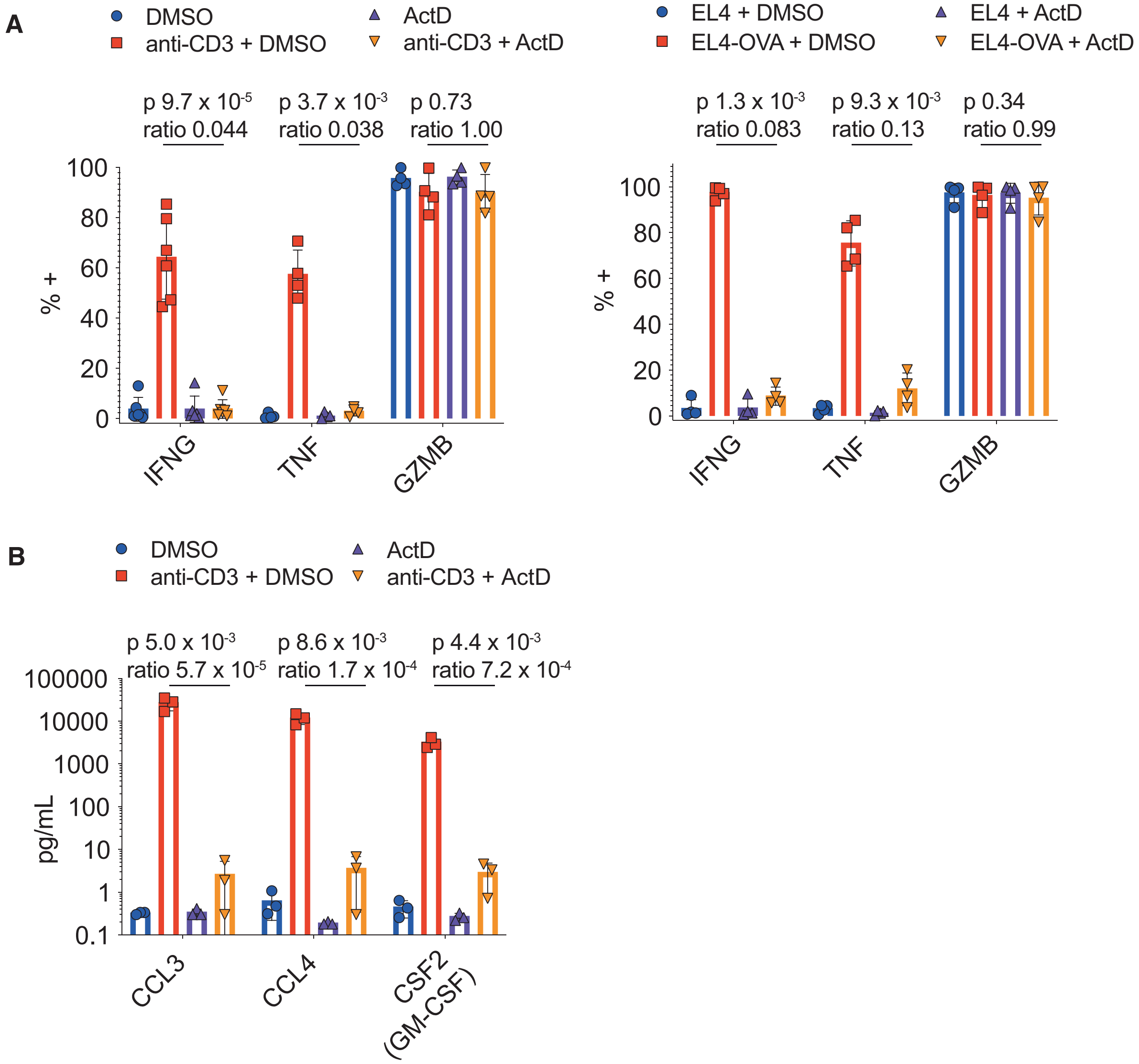

Figure EV2. Actinomycin D treatment blocks stimulation-induced production of cytokines/chemokines at 4 h.

Flow cytometry and multiplexed cytokine/chemokine bead assays were used to examine the impact of actinomycin D (ActD) treatment on the CTL secretome.

A OTI CTLs were stimulated with or without plate-bound anti-CD3 (left), or antigen-pulsed versus unpulsed EL4 target cells (EL4-OVA or EL4, respectively, right), in the presence of ActD or DMSO vehicle control, for 4 h. IFNG, TNF, and GZMB expression were measured by flow cytometry. For IFNG and TNF staining, secretion was blocked during TCR stimulation to allow intracellular cytokine staining. Bar plots summarise results from six biological replicates per measurement for IFNG under anti-CD3 stimulation, and four biological replicates for all other measurements.

B Supernatants were collected from OTI CTLs stimulated with or without plate-bound anti-CD3, in the presence of DMSO or ActD, for 4 h, and chemokine secretion was measured by multiplexed bead assay. Plot summarises results from three biological replicates.

Data information: (A, B) Bar heights and error bars depict means and standard deviations, respectively. Points show individual biological replicates. *P*-values by ratio paired *t*-tests comparing stimulated CTLs treated with ActD or DMSO.

 © 2023 The Authors

Figure EV3. Stimulation-induced upregulation of *Gzmb* and *Prf1* mRNA is impaired by actinomycin D but degranulation and cytolytic functions are retained for several hours.

A *Gzmb* and *Prf1* mRNA expression was measured by RNA flow cytometry in CTLs challenged at a 1:1 ratio with antigen-pulsed versus unpulsed EL4 target cells (EL4-OVA or EL4, respectively), in the presence of actinomycin D (ActD) or DMSO control, for 2 and 4 h. Cell-based histograms (left) show fluorescence of each marker in one biological replicate, representative of 3. Lines indicate the threshold for positive staining. Bar plots (right) show median fluorescent intensity of mRNA stains among all CTLs in each condition, compiling results from all biological replicates. Bar heights depict means and points show individual biological replicates. *P*-values by ratio paired *t*-test comparing stimulated CTLs treated with ActD or DMSO.

B Degranulation was measured in OTI CTLs challenged as in (A) at a 1:1 CTL:target ratio for 3 h. Histograms (left) depict staining of LAMP1, which is exposed on the CTL surface as cytolytic granules are released, on one biological replicate, representative of 5. Line indicates gate used to delineate degranulated cells. Plot (right) depicts combined data from all biological replicates with values from the same replicate connected by a line (note two replicates are nearly identical and thus not visually separated on plot); *P*-values by ratio paired *t*-test.

C To test the impact of ActD on target cell viability and growth, DMSO or ActD was added to red EL4-OVA in the absence of any CTLs and red fluorescent intensity monitored by live imaging over time. Points and error bars depict the means and standard deviations of three technical replicates for each measurement. Results are representative of two experimental repeats performed 1 month apart with this cell line.

D CTLs were challenged as in (A) for 4 h by mixing with target cells at the indicated CTL:target ratios, and target cell lysis measured by release of lactate dehydrogenase (LDH) into the media. Plot depicts results from four biological replicates with lines connecting treatments within each replicate for each ratio. Statistical analysis was performed as a 2-way ANOVA with Geisser–Greenhouse correction on log-transformed data to compare ActD/DMSO cytolysis between treatments; *P*-values are reported for "treatment" and "treatment × CTL:target ratio interaction" terms. The ratio of target cell lysis with ActD versus DMSO treatment is also reported for each CTL:target ratio.

© 2023 The Authors

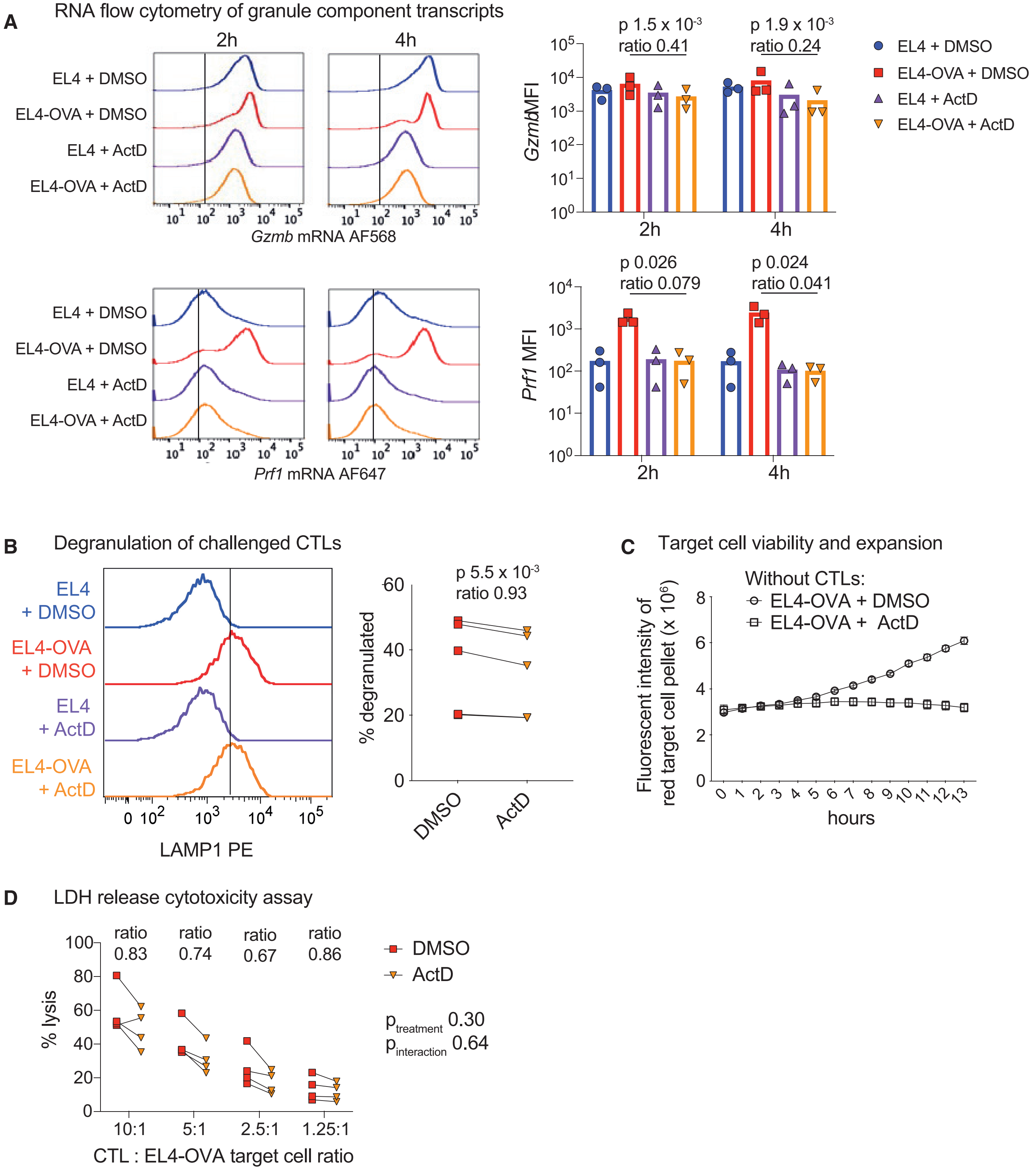

Figure EV3.

© 2023 The Authors

Figure EV4. Actinomycin D treatment does not alter basal CTL migration speed.

CTLs were pre-treated with actinomycin D (ActD) or DMSO vehicle control and filmed 3–5 h later migrating on ICAM-1-coated glass.

A Images depict the maximum projection of z-stacks from the last image of representative movies, with nuclei labelled with spheres and the tracks followed by the migrating CTLs depicted in rainbow; green, CFSE; blue, Hoechst. Representative of 2–5 movies (total 144–422 cells) per condition from each of two biological replicates. Scale bars (white, lower left) = 30 μm.

B Quantification of track mean speed and track straightness for all cells filmed from one biological replicate.

C Non-parametric Hodges-Lehmann estimate and 95% confidence interval for difference between ActD and DMSO treatment for parameters in (B) in each of the two biological replicates.

© 2023 The Authors

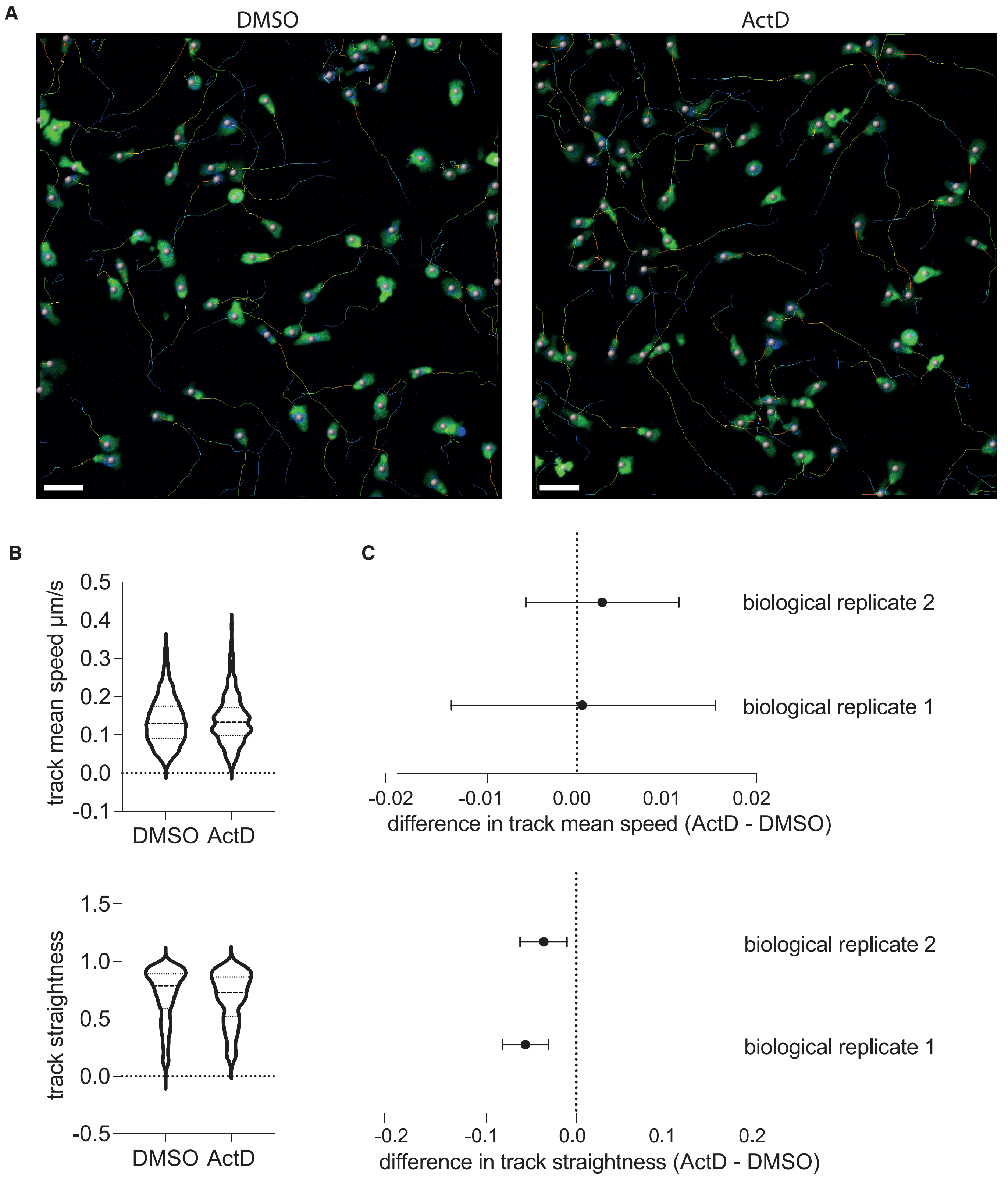

Figure EV4.

 © 2023 The Authors

Figure EV5. Characterisation of *Rab27a/b*$^{-/-}$ OTI CTLs.

A Degranulation was measured in WT OTI and RAB27A/B-deficient OTI CTLs challenged with antigen-pulsed versus unpulsed EL4 target cells (EL4-OVA or EL4, respectively) for 3 h. Histograms depict staining of LAMP1, which is exposed on the CTL surface as cytolytic granules are released. Results are representative of three biological replicates of each genotype.

B WT OTI or RAB27A/B-deficient OTI CTLs were layered on top of EL4-OVA or EL4 target cells expressing a red fluorescent nuclear protein to measure cytolytic activity through live imaging. Points and error bars depict the means and standard deviations of three technical replicates for each measurement within one biological replicate, representative of 3. Negative death reflects the growth of target cells during the assay.

C, D WT OTI and RAB27A/B-deficient OTI CTLs were challenged (C) with or without plate-bound anti-CD3, or (D) with EL4-OVA or EL4 target cells for 4 h before measuring cytokine expression by flow cytometry. Bar plots (right) depict compiled results from five biological replicates of each genotype. Bar heights and error bars depict means and standard deviations, respectively. Points show individual biological replicates.

E Supernatants collected from CTLs challenged as in (C) were assayed for cytokine secretion. Bar plots summarise results from four biological replicates of each genotype. Bar heights depict means and points show individual biological replicates.

Data information: *P*-values (C–E) by Welch's *t*-test compare stimulated WT with stimulated RAB27A/B-deficient CTLs.

© 2023 The Authors

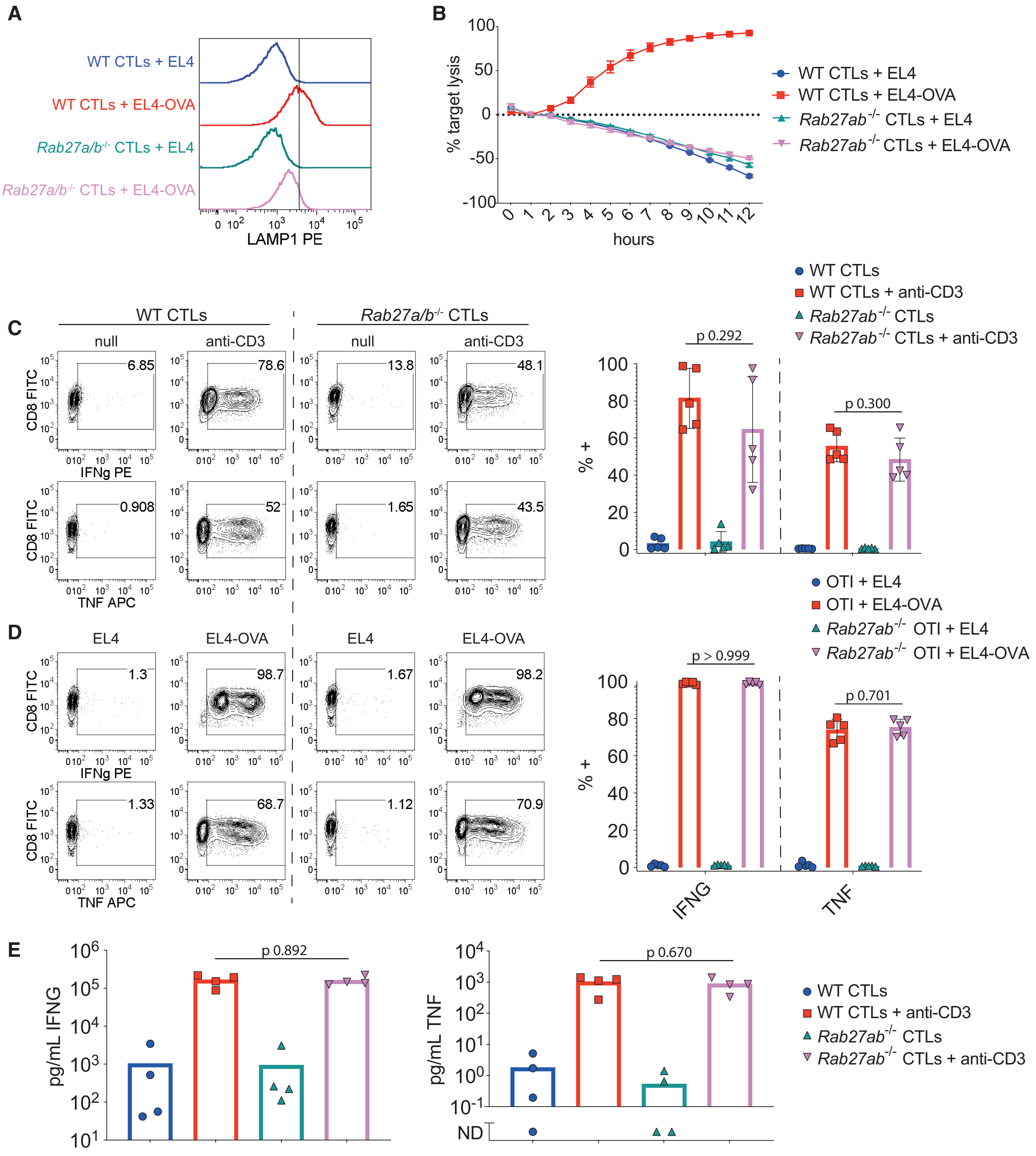

Figure EV5.

 © 2023 The Authors
