# Supplementary material for: Cytotoxic T lymphocytes require transcription for infiltration but not target cell lysis
Source: EMBO Rep. 2023 Oct 20;24(11):e57653. doi: 10.15252/embr.202357653 (PMC10626425; doi:10.15252/embr.202357653)
Supplement: Supplementary file 12 [file EMBR-24-e57653-s011.pdf]

EMBO reports

Cytotoxic T lymphocytes require transcription for infiltration but not target cell lysis

Arianne Richard, Claire Ma, John Marioni and Gillian Griffiths
DOI: 10.15252/embr.202357653

Corresponding author(s): Gillian Griffiths (gg305@cam.ac.uk)

Review Timeline:

Transfer Date:	22nd Jun 23
Editorial Decision:	25th Jul 23
Revision Received:	24th Aug 23
Editorial Decision:	11th Sep 23
Revision Received:	19th Sep 23
Accepted:	25th Sep 23

Editor: Achim Breiling

Transaction Report: A revised version of this manuscript was transferred to EMBO *reports* following peer review at the EMBO Journal.

Referee #1 Review

Received: 13th Sep 22

Report for Author:

Richard et al have investigated the requirement for de novo transcription in the execution of effector functions by in-vitro differentiated cytotoxic T lymphocytes (CTLs). Using actinomycin D, a drug that blocks RNA synthesis, the researchers have made the observation that the proteins that are highly upregulated upon TCR signaling (chemokines, IFNg, TNF, and transcriptional regulators) are dependent upon transcription. Interestingly, they report that the proteins involved in target cell lysis stay largely unchanged in abundance upon TCR engagement and are relatively unaffected by transcriptional blockade. They further show that this correlates well in functional studies where actinomycin D treated CTLs mixed with target cells are able to lyse their targets at rates similar to transcription competent CTLs. However, actinomycin D treated CTLs were inept at creating a chemotaxis gradient for other CTLs and had impeded infiltration capacity when layered on top of a pellet of target cells. These experiments demonstrate that key effector functions in CTLs are regulated at the transcriptional level, providing avenues for modulation in therapy of autoimmune diseases and cancer.

The manuscript boasts clear writing with precise conclusions backed by rigorous data. The authors have leveraged their expertise with live-cell imaging to produce convincing functional data for CTLs. However, it is not immediately apparent how this work adds to the current understanding of T-cell biology. The questions asked relate to transcription in general, making the conclusions one can draw very broad. It is not surprising that CTLs require transcription for making cytokines, or that a reserve of preformed granules can be mobilized for target cell lysis even when RNA synthesis is blocked. While there are some interesting conclusions to be drawn from the data, in the absence of additional mechanistic information (such as identifying specific transcription factors, chemotaxis signaling proteins, etc.) to tie together the functional consequences described, the overall impact of the studies presented is modest.

Minor comment:

1. Figure 5A would benefit from the use of more easily distinguishable symbols for the different experimental conditions shown.

Referee #2 Review

Received: 21st Sep 22

Report for Author:

The paper addresses the role of transcription in the function of cytotoxic T cells (CTL). OT-I were stimulated with 10 nM SIINFEKL peptide for 3 days and used on days 6-8 after initial stimulation. Such in vitro-derived effector CTLs were stimulated for 0, 10, 20, 40 and 60 minutes with anti- CD3 and subjected to RNA-seq. Expression of Tnf and of Egr1 and Egr2 was upregulated 20 minutes after stimulation. 60 minutes after stimulation, upregulated gene comprised cytokines (ifng) and chemokines (ccl23) whereas the expression of genes encoding the cytotoxic proteins granzymes and perforin showed no change. Mass spectrometry was used next to profile the proteome of effector CTLs at rest and after 4 hours of stimulation with anti-CD3, in the presence or absence of actinomycin D, which blocks RNA synthesis. Proteins affected by actinomycin D only in the context of TCR stimulation included cytokines (IFNG, XCL1, CCL3, CCL1, CSF2), IEGs (NR4A2, NR4A3, EGR1, EGR2), and transcription factors known to drive CTL differentiation (TBX21 and EOMES). Analysis of the relationship between transcriptomic and proteomic data, collected after 1 and 4 hours of TCR stimulation suggested that the most extreme changes in protein expression 4 hours after TCR stimulation likely rely on transcription and not simply translation of pre-existing mRNAs. Those data were corroborated by cytofluorometric analysis and extended to challenge with EL4 antigen-loaded target cells. Using live imaging to monitor the death of fluorescent target cells over time, CTLs persisted in a state poised for cytolytic activity for several hours. Using an assay imaging CTL infiltration (see major comment below), RAB27A/B-deficient CTLs were capably of infiltrating the target cell pellet prior to actinomycin D treatment. Finally, by exploiting RAB27A/B-deficient CTLs as a source of chemotactic signals, the authors showed that after co-culture with transcription-inhibited wild-type CTLs, they improved target pellet infiltration, leading to the interesting conclusion that infiltration and subsequent target cell lysis require transcription by the infiltrating CTL. Provided that the major comment specified below is properly addressed, the conclusions of the paper are of interest for immunologists and might also fit in EMBO Reports.

Major comment.
The most interesting part of the paper corresponds to the last two paragraphs and is intended to demonstrate that both generation of chemotactic signals and infiltration among target cells require transcription. I have however difficulties to understand the way CTL infiltration was assessed/monitored and a scheme will be appreciated. The authors indicate in the Material and Methods that CTLs were layered on top of an EL4 target cell pellet and image using an IncuCyte S3 Live Cell Analysis System. How thick and how compact is the EL4 target cell pellet? How is the pellet formed and how its thickness and compactness are controlled? How does it reflect an infected tissue or a tumor microenvironment? How does it compare to three recent reports (https://doi.org/10.1172/JCI159402, DOI: 10.1126/sciimmunol.abd4344 and the solid tumouroids embedded in three- dimensional collagen matrices used in Ref. 16) that appeared to use experimental set up that more closely mimic in vivo tissue conditions ? Moreover, no rational is given for the fact that 'the area of the dish occupied by CTLs contracts as they migrate toward and infiltrate the target cell pellet', a key feature used for quantifying the data.
Minor comments
1/ Page 11: concentration of Actinomycin D is missing.
2/ Page 4: programs instead of programmes

Referee #3 Review
Received: 1st Oct 22

Report for Author:
Activated CTL kill antigenic target cells using preformed lytic granules and further undergo restimulation followed by transcription, cytokine release and re-onset of proliferation. Which components of this effector cycle depends on de-novo transcription and which functions can be executed from protein and RNA stores remains unclear.
Richard and co-workers performed time-gated bulk RNAseq and proteome analysis to test the transcriptional changes induced by TCR re-stimulation in recently activated OT-1 CTL and the consequences of transcription for effector function and recruitment. The data show convincingly, that cytolytic protein expression and very early cytolytic activity resist transcriptional blockade, whereas cytokine and chemokine production depend on TCR-signal induced de-novo transcription. Using in vitro coculture assays, they further show that transcription is required for hetero- and homotypic aggregation, as well as chemotaxis induction in trnswell assays. Lastly, RAB27A/B-deficient CTLs were used to discriminate effects caused by cytotoxic granule release from RAB27A/B-independent cytokine release.

The time-gated correlative analysis of altered gene expression (transcriptome) with protein (proteome), the dependence of ffects on Rab27A/B, and the chemotaxis data are convincing. However, the claim, initial that CTL-mediated killing does not depend on de-novo transcription, is underpowered, because the time-dependent functional analyses were performed at an excessively high E:T ratio (10:1). The results from the CTL-EL4 aggregation assay does not conclusively support the claim of CTL infiltration into the tumor compartment. In addition, several shortcomings on data reporting should be revised.

1. The effect of ActD on target cell killing is not well represented, across independent experiments and lack statistical analysis. Which portion of killing depends on de-novo synthesis of granule content, for example after 1h versus 4 h of inhibition of

translation? The effect is likely gradual and may be visible only when the E:T ratio is not totally overloaded (as in Fig. 3C, E:T = 10:1 !), where even the endpoints after 10 hours do not differ. To support the claim that early killing does not depend on rapid refilling (by translation) of granule content, more subtle analysis on the time-dependence needs to be shown, such as using E:T ratios of 1:1 or 1:8. Because the process is likely gradual, more fine-grained analysis of the decrease of efficacy per CTL should be provided, using single-cell monitoring.
In addition, related to Figs. 4B&C, 5A, to fulfil standards of good scientific reporting and in order to perform the statistics necessary to support the claims (discriminating near-undiminished from diminishing killing ability), the data need to show the means and SD of independent experiments (currently only triplicate data of one representative experiment without statistics is shown).

2. Fig. 5B and Movies 1-3: it is not clear which cell functions this complex assay represents. The authors claim, that CRL infiltration (red) into target cell layers is shown, however from the videos it is apparent that the target cell cluster aggregates with the CTL and contracts, to form a concentric and optically dense ball and that even outer target cell regions which do not engage with CTL directly seem to contract with their neighbors. EL4 cells are B cell derived, hence bystander activation of B cell aggregation may be triggered, which very likely convolutes the readout. Thus, the claim of CTL recruitment is not well supported by the assay and the interpretation of the biological process should be reconsidered.
The related data reporting on "cytolytic activity" (legend Fig. 4B) is not clear either. What does "% bright field area compared to 1h represent". The entire cluster area? The authors state: "The area of the well occupied by cells was quantified at 5 hours", hance likely B cell aggregation ws included in the readout. In addition, it would be helpful so show primary images (unless they are the same as in Fig. 5C). It is further not clear, why the data points are connected, assuming that control and ActD values were obtained from independent wells.
To support the claim, CTL migration into a pre-aggregated target cell tumoroid should be shown, monitored by serial sectioning at the endpoint or live-cell recording and tracking.

3. Fig. 4D: The plots lack a (negative) baseline control, e.g. using naïve OT1-CTL. To compare the result to baseline expression of granzyme B and perforin, as an estimate of CTL largely depleted of cytolytic granules, e.g. unstimulated OT-1 CTL should be included in this panel.

4. The inability of cytotoxic activity of Rab27A/B-deficient OT1-CTL should be shown for their model system.

Minor points

Fig. S1B: It is not clear where viability is depicted. The number of "morphologically intact" cells may be misleading given the short time period of incubation and follow-up. Please add quantification of live-dead dye staining (e.g., propidium iodide) and/or, more sensitive, annexin-V detection of damaged cells.

Fig. 4A: the gating strategy should be shown. Were PI- or annexin-V positive cells excluded, to rule out false-positive events? This is potentially critical, because the lysis ability (shown in Fig. 4B, C) is somewhat diminished after treatment with ActD.

Fig. 4D and: The means (D) and densitometric analyses from n=3 should be included, as well as statistical analysis.

We thank the reviewers for their comments. In revising the manuscript, we have addressed the points that they raised as detailed in the responses below in blue, with relevant sections indicated by blue font in the attached manuscript.

Response to Reviewers:

Referee #1:

Richard et al have investigated the requirement for de novo transcription in the execution of effector functions by in-vitro differentiated cytotoxic T lymphocytes (CTLs). Using actinomycin D, a drug that blocks RNA synthesis, the researchers have made the observation that the proteins that are highly upregulated upon TCR signaling (chemokines, IFNg, TNF, and transcriptional regulators) are dependent upon transcription. Interestingly, they report that the proteins involved in target cell lysis stay largely unchanged in abundance upon TCR engagement and are relatively unaffected by transcriptional blockade. They further show that this correlates well in functional studies where actinomycin D treated CTLs mixed with target cells are able to lyse their targets at rates similar to transcription competent CTLs. However, actinomycin D treated CTLs were inept at creating a chemotaxis gradient for other CTLs and had impeded infiltration capacity when layered on top of a pellet of target cells. These experiments demonstrate that key effector functions in CTLs are regulated at the transcriptional level, providing avenues for modulation in therapy of autoimmune diseases and cancer.
The manuscript boasts clear writing with precise conclusions backed by rigorous data. The authors have leveraged their expertise with live-cell imaging to produce convincing functional data for CTLs. However, it is not immediately apparent how this work adds to the current understanding of T-cell biology. The questions asked relate to transcription in general, making the conclusions one can draw very broad. It is not surprising that CTLs require transcription for making cytokines, or that a reserve of preformed granules can be mobilized for target cell lysis even when RNA synthesis is blocked. While there are some interesting conclusions to be drawn from the data, in the absence of additional mechanistic information (such as identifying specific transcription factors, chemotaxis signaling proteins, etc.) to tie together the functional consequences described, the overall impact of the studies presented is modest.

We appreciate that the reviewer found the writing, conclusions and data both clear and convincing. We believe that we do add to the fundamental understanding of how CTLs function through two important findings:

1) The functional consequences of TCR engagement on effector CTLs can be separated by two fundamentally different biosynthetic mechanisms, those dependent on transcription (secretion of cytokines/chemokines and infiltration), and those that occur independently of de novo RNA synthesis (cytolytic granule release and lysis of neighbouring target cells). Previous studies identified different expression of individual proteins that fall into these functional categories, but our comprehensive description of this phenomenon and its generalizability is new.
2) CTL infiltration is dependent on transcription. This has never before been described.

However, we realize that these messages may have been watered-down by presenting them among other findings that were less clear or novel. We have reorganised the manuscript to focus on the novel findings and move supporting but less novel data to the supplement. We have also reformatted some data to be as clear as possible in our messages.

Minor comment:
1. Figure 5A would benefit from the use of more easily distinguishable symbols for the different experimental conditions shown.

These have been changed.

Referee #2:

The paper addresses the role of transcription in the function of cytotoxic T cells (CTL). OT-I were stimulated with 10 nM SIINFEKL peptide for 3 days and used on days 6-8 after initial stimulation. Such in vitro-derived effector CTLs were stimulated for 0, 10, 20, 40 and 60 minutes with anti- CD3 and subjected to RNA-seq. Expression of Tnf and of Egr1 and Egr2 was upregulated 20 minutes after stimulation. 60 minutes after stimulation, upregulated gene comprised cytokines (ifng) and chemokines (ccl23) whereas the expression of genes encoding the cytotoxic proteins granzymes and perforin showed no change. Mass spectrometry was used next to profile the proteome of effector CTLs at rest and after 4 hours of stimulation with anti-CD3, in the presence or absence of actinomycin D, which blocks RNA synthesis. Proteins affected by actinomycin D only in the context of TCR stimulation included cytokines (IFNG, XCL1, CCL3, CCL1, CSF2), IEGs (NR4A2, NR4A3, EGR1, EGR2), and transcription factors known to drive CTL differentiation (TBX21 and EOMES). Analysis of the relationship between transcriptomic and proteomic data, collected after 1 and 4 hours of TCR stimulation suggested that the most extreme changes in protein expression 4 hours after TCR stimulation likely rely on transcription and not simply translation of pre-existing mRNAs. Those data were corroborated by cytofluorometric analysis and extended to challenge with EL4 antigen-loaded target cells. Using live imaging to monitor the death of fluorescent target cells over time, CTLs persisted in a state poised for cytolytic activity for several hours. Using an assay imaging CTL infiltration (see major comment below), RAB27A/B-deficient CTLs were capably of infiltrating the target cell pellet prior to actinomycin D treatment. Finally, by exploiting RAB27A/B-deficient CTLs as a source of chemotactic signals, the authors showed that after co-culture with transcription-inhibited wild-type CTLs, they improved target pellet infiltration, leading to the interesting conclusion that infiltration and subsequent target cell lysis require transcription by the infiltrating CTL. Provided that the major comment specified below is properly addressed, the conclusions of the paper are of interest for immunologists and might also fit in EMBO Reports.

Major comment.
The most interesting part of the paper corresponds to the last two paragraphs and is intended to demonstrate that both generation of chemotactic signals and infiltration among target cells require transcription. I have however difficulties to understand the way CTL infiltration was assessed/monitored and a scheme will be appreciated. The authors indicate in the Material and Methods that CTLs were layered on top of an EL4 target cell pellet and image using an IncuCyte S3 Live Cell Analysis System. How thick and how compact is the EL4 target cell pellet? How is the pellet formed and how its thickness and compactness are controlled? How does it reflect an infected tissue or a tumor microenvironment? How does it compare to three recent reports (https://doi.org/10.1172/JCI159402, DOI: 10.1126/sciimmunol.abd4344 and the solid tumouroids embedded in three- dimensional collagen matrices used in Ref. 16) that appeared to use experimental set up that more closely mimic in vivo tissue conditions ? Moreover, no rational is given for the fact that 'the area of the dish occupied by CTLs contracts as they migrate toward and infiltrate the target cell pellet', a key feature used for quantifying the data.

We appreciate that the reviewer found our results of potential interest. The description of the infiltration assay has been elaborated in the Methods. In the discussion, we have also commented on how it compares to infiltration assays in [Galeano Nino, *et al.* 2020 Elife]. The first paper suggested by the reviewer [Romain, *et al.* 2022 JCI] focuses on migration instead of infiltration, which we have ruled out in Supplemental Figure 4. The second suggested paper uses *in vivo* investigation to examine how infiltrating CAR T cells modify the tumour microenvironment (TME), highlighting the role of IFNg in sustaining cytotoxicity by interplaying with host cells in the TME. We have included this reference in the Discussion while highlighting the caveats of a model that uses only tumour target cells and CTLs. Although our co-culture experiment using RAB27A/B-deficient CTLs to supply a gradient of exogenous cytokines/chemokine (now Figure 4E-F) includes IFNG, for completeness we have also tested direct addition of IFNG in a cytotoxicity assay that requires CTL infiltration of a target cell pellet (Figure for Reviewers). This demonstrated no effect of exogenous IFNG, indicating that it is not responsible for defective infiltration/killing by transcription-inhibited CTLs.

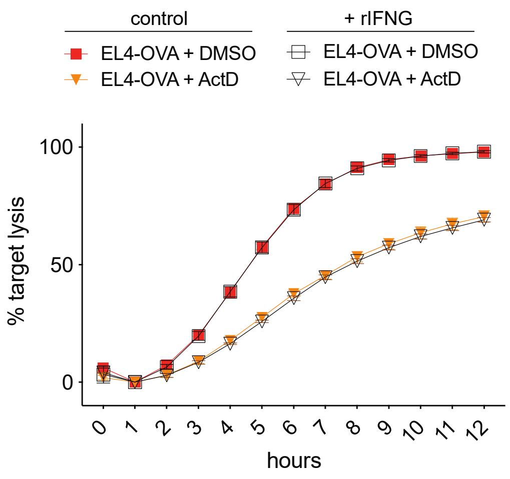

Figure for Reviewers: *Exogenous IFNG does not impact target cell killing when CTLs must infiltrate the target cell pellet. Cytolytic activity was measured in real time by live cell imaging of WT OTI CTLs layered on top of antigen-pulsed versus unpulsed red EL4 target cells (EL4-OVA or EL4, respectively), in the presence of actinomycin D (ActD) or DMSO vehicle control, without (coloured shapes) or with (open shapes) the addition of 100 ng/mL recombinant murine IFNG. Points and error bars indicate the mean and standard deviation of 3 technical replicates per measurement. Results are representative of 2 biological replicates.*

Minor comments
1/ Page 11: concentration of Actinomycin D is missing.

The concentration (5 ug/mL) of actinomycin D was included on original p 11, but we have now repeated it later in the paragraph for clarity.

2/ Page 4: programs instead of programmes

This has been fixed.

Referee #3:

Activated CTL kill antigenic target cells using preformed lytic granules and further undergo restimulation followed by transcription, cytokine release and re-onset of proliferation. Which components of this effector cycle depends on de-novo transcription and which functions can be executed from protein and RNA stores remains unclear.
Richard and co-workers performed time-gated bulk RNAseq and proteome analysis to test the transcriptional changes induced by TCR re-stimulation in recently activated OT-1 CTL and the consequences of transcription for effector function and recruitment. The data show convincingly, that cytolytic protein expression and very early cytolytic activity resist transcriptional blockade, whereas cytokine and chemokine production depend on TCR-signal induced de-novo transcription. Using in vitro coculture assays, they further show that transcription is required for hetero- and homotypic aggregation, as well as chemotaxis induction in trnswell assays. Lastly, RAB27A/B-deficient CTLs were used to discriminate effects caused by cytotoxic granule release from RAB27A/B-independent cytokine release.

The time-gated correlative analysis of altered gene expression (transcriptome) with protein (proteome), the dependence of ffects on Rab27A/B, and the chemotaxis data are convincing. However, the claim, initial that CTL-mediated killing does not depend on de-novo transcription, is underpowered, because the time-dependent functional analyses were performed at an excessively high E:T ratio (10:1). The results from the CTL-EL4 aggregation assay does not conclusively support the claim of CTL infiltration into the tumor compartment. In addition, several shortcomings on data reporting should be revised.

1. The effect of ActD on target cell killing is not well represented, across independent experiments and lack statistical analysis. Which portion of killing depends on de-novo synthesis of granule content, for example after 1h versus 4 h of inhibition of translation? The effect is likely gradual and may be visible only when the E:T ratio is not totally overloaded (as in Fig. 3C, E:T = 10:1 !), where even the endpoints after 10 hours do not differ. To support the claim that early killing does not depend on rapid refilling (by translation) of granule content, more

subtle analysis on the time-dependence needs to be shown, such as using E:T ratios of 1:1 or 1:8. Because the process is likely gradual, more fine-grained analysis of the decrease of efficacy per CTL should be provided, using single-cell monitoring.

There appears to have been a misunderstanding and perhaps some confusion about our claims regarding transcription versus translation. Our live-imaging assay was not intended to test granule refilling. Instead, we were simply looking at target killing over a long time-period after transcription blockade. In retrospect, the description of this figure was confusing and veered off point. We have therefore moved data examining de novo transcription of cytolytic proteins to the supplement and removed most of the speculative discussion about granule refilling as this data is not conclusive (as discussed) and not the main focus of the manuscript.

In addition, related to Figs. 4B&C, 5A, to fulfil standards of good scientific reporting and in order to perform the statistics necessary to support the claims (discriminating near-undiminished from diminishing killing ability), the data need to show the means and SD of independent experiments (currently only triplicate data of one representative experiment without statistics is shown).

We have now included summary data for all replicates and statistics to these and other figures.

2. Fig. 5B and Movies 1-3: it is not clear which cell functions this complex assay represents. The authors claim, that CRL infiltration (red) into target cell layers is shown, however from the videos it is apparent that the target cell cluster aggregates with the CTL and contracts, to form a concentric and optically dense ball and that even outer target cell regions which do not engage with CTL directly seem to contract with their neighbors. EL4 cells are B cell derived, hence bystander activation of B cell aggregation may be triggered, which very likely convolutes the readout. Thus, the claim of CTL recruitment is not well supported by the assay and the interpretation of the biological process should be reconsidered.

Unfortunately, there has been a misunderstanding here. The target cells were red, not the infiltrating CTLs. This led to a misinterpretation of the data and confusion about our conclusion. We have clarified this in the revised manuscript.

The related data reporting on "cytolytic activity" (legend Fig. 4B) is not clear either.

This has been clarified with reorganisation and revised text.

What does "% bright field area compared to 1h represent". The entire cluster area? The authors state: "The area of the well occupied by cells was quantified at 5 hours", hance likely B cell aggregation ws included in the readout. In addition, it would be helpful so show primary images (unless they are the same as in Fig. 5C). It is further not clear, why the data points are connected, assuming that control and ActD values were obtained from independent wells.
To support the claim, CTL migration into a pre-aggregated target cell tumoroid should be shown, monitored by serial sectioning at the endpoint or live-cell recording and tracking.

This criticism stems back to the misunderstanding of which cells are CTLs and which are tumour targets, which we have now clarified. Primary images and live-cell recording are already included in the figure. Connected lines represent cells from the same mouse, which is now described in the legend.

3. Fig. 4D: The plots lack a (negative) baseline control, e.g. using naïve OT1-CTL. To compare the result to baseline expression of granzyme B and perforin, as an estimate of CTL largely depleted of cytolytic granules, e.g. unstimulated OT-1 CTL should be included in this panel.

This figure panel examines RNA expression, not protein, and thus a CTL depleted of cytolytic granules (i.e. of proteins) is not an appropriate control. Likewise, a naïve CD8+ T cell is of an entirely different cell biological and metabolic state and is not appropriate. Unstimulated effector CTLs are included in the absence of OVA antigen (EL4 + DMSO, and EL4 + ActD conditions) as the appropriate controls. Gating, depicted in Supplemental Figure 6B (previously Supplemental Figure 4B) was performed using fluorescence-minus-one (FMO) controls to represent a true negative, as is standard in the field.

4. The inability of cytotoxic activity of Rab27A/B-deficient OT1-CTL should be shown for their model system.

This was already included in Supplemental Figure 5B (previously Supplemental Figure 3B) but has been reformatted for ease of understanding.

Minor points

Fig. S1B: It is not clear where viability is depicted. The number of "morphologically intact" cells may be misleading given the short time period of incubation and follow-up. Please add quantification of live-dead dye staining (e.g., propidium iodide) and/or, more sensitive, annexin-V detection of damaged cells.

This is not a plot of morphology as the reviewer suggests. The LD aqua stain depicted on the y-axis is a live-dead stain (Zombie, Biolegend). This has been clarified in the figure legend.

Fig. 4A: the gating strategy should be shown. Were PI- or annexin-V positive cells excluded, to rule out false-positive events? This is potentially critical, because the lysis ability (shown in Fig. 4B, C) is somewhat diminished after treatment with ActD.

The gating strategy is already shown in Supplemental Figure 6C (previously Supplemental Figure 4C) and does include a live-dead stain.

Fig. 4D and: The means (D) and densitometric analyses from n=3 should be included, as well as statistical analysis.

Total perforin levels depicted in this Western Blot were already captured in the mass spectrometry analysis with accompanying statistical information (Figure 2 and Supplemental Table 2). In simplifying the manuscript to focus on key messages, we have removed this panel.

1st Editorial Decision 25th Jul 2023

Dear Prof. Griffiths

Thank you for the transfer of your revised manuscript to EMBO reports. I have now received the reports from the three referees that were asked to re-evaluate your study (the same that have assessed the work for The EMBO Journal), you will find below. As you will see, referees #1 and #2 now fully support publication of your study in EMBO reports. Referee #3 has remaining concerns I ask you to address in a final revised manuscript. Please also provide a final p-b-p-response addressing these points. Finally, please note the suggestion of referee #1.

Moreover, the manuscript needs also formatting according to our journal style. Please carefully review the instructions that follow below.

PLEASE NOTE THAT upon resubmission revised manuscripts are subjected to an initial quality control prior to exposition to re-review. Upon failure in the initial quality control, the manuscripts are sent back to the authors, which may lead to delays. Frequent reasons for such a failure are the lack of the data availability section (please see below) and the presence of statistics based on n=2 (the authors are then asked to present scatter plots or provide more data points).

When submitting your revised manuscript, we will require:

1) a .docx formatted version of the final manuscript text (including legends for main figures, EV figures and tables), but without the figures included. Figure legends should be compiled at the end of the manuscript text.

2) individual production quality figure files as .eps, .tif, .jpg (one file per figure), of main figures (up to 8) and EV figures (up to 5). Please upload these as separate, individual files upon re-submission.

The Expanded View format, which will be displayed in the main HTML of the paper in a collapsible format, has replaced the Supplementary information. You can submit up to 5 images as Expanded View. Please follow the nomenclature Figure EV1, Figure EV2 etc. The figure legend for these should be included in the main manuscript document file in a section called Expanded View Figure Legends after the main Figure Legends section. Additional Supplementary material should be supplied as a single pdf file labeled Appendix. The Appendix should have page numbers and needs to include a table of content on the first page (with page numbers) and legends for all content. Please follow the nomenclature Appendix Figure Sx, Appendix Table Sx etc. throughout the text, and also label the figures and tables according to this nomenclature.

For more details, please refer to our guide to authors:
http://www.embopress.org/page/journal/14693178/authorguide#manuscriptpreparation

Please consult our guide for figure preparation:
http://wol-prod-cdn.literatumonline.com/pb-assets/embo-site/EMBOPress_Figure_Guidelines_061115-1561436025777.pdf

See also the guidelines for figure legend preparation:
https://www.embopress.org/page/journal/14693178/authorguide#figureformat

3) a complete author checklist, which you can download from our author guidelines (https://www.embopress.org/page/journal/14693178/authorguide). Please insert page numbers in the checklist to indicate where the requested information can be found in the manuscript. The completed author checklist will also be part of the RPF.

Please also follow our guidelines for the use of living organisms, and the respective reporting guidelines:
http://www.embopress.org/page/journal/14693178/authorguide#livingorganisms

4) that primary datasets produced in this study (e.g. RNA-seq, ChIP-seq, structural and array data) are deposited in an appropriate public database. If no primary datasets have been deposited, please also state this in a dedicated section (e.g. 'No primary datasets have been generated and deposited'), see below.

See also: http://embor.embopress.org/authorguide#datadeposition

Please remember to provide a reviewer password if the datasets are not yet public.

The accession numbers and database should be listed in a formal "Data Availability" section (placed after Materials & Methods) that follows the model below. This is now mandatory (like the COI statement). Please note that the Data Availability Section is restricted to new primary data that are part of this study. This section is mandatory. As indicated above, if no primary datasets have been deposited, please state this in this section

Data availability

The datasets produced in this study are available in the following databases:

- RNA-Seq data: Gene Expression Omnibus GSE46843 (https://www.ncbi.nlm.nih.gov/geo/query/acc.cgi?acc=GSE46843)
- [data type]: [name of the resource] [accession number/identifier/doi] ([URL or identifiers.org/DATABASE:ACCESSION])

*** Note - All links should resolve to a page where the data can be accessed. ***

Moreover, I have these editorial requests:

5) We now request the publication of original source data with the aim of making primary data more accessible and transparent to the reader. Our source data coordinator will contact you to discuss which figure panels we would need source data for and will also provide you with helpful tips on how to upload and organize the files.

6) Our journal encourages inclusion of *data citations in the reference list* to directly cite datasets that were re-used and obtained from public databases. Data citations in the article text are distinct from normal bibliographical citations and should directly link to the database records from which the data can be accessed. In the main text, data citations are formatted as follows: "Data ref: Smith et al, 2001" or "Data ref: NCBI Sequence Read Archive PRJNA342805, 2017". In the Reference list, data citations must be labeled with "[DATASET]". A data reference must provide the database name, accession number/identifiers and a resolvable link to the landing page from which the data can be accessed at the end of the reference. Further instructions are available at: http://www.embopress.org/page/journal/14693178/authorguide#referencesformat

7) Regarding data quantification and statistics, please make sure that the number "n" for how many independent experiments were performed, their nature (biological versus technical replicates), the bars and error bars (e.g. SEM, SD) and the test used to calculate p-values is indicated in the respective figure legends (also for potential EV figures and all those in the final Appendix). Please also check that all the p-values are explained in the legend, and that these fit to those shown in the figure. Please provide statistical testing where applicable. Please avoid the phrase 'independent experiment', but clearly state if these were biological or technical replicates. Please also indicate (e.g. with n.s.) if testing was performed, but the differences are not significant. In case n=2, please show the data as separate datapoints without error bars and statistics. See also: http://www.embopress.org/page/journal/14693178/authorguide#statisticalanalysis

If n<5, please show single datapoints for diagrams.

8) Please add scale bars of similar style and thickness to microscopic images, using clearly visible black or white bars (depending on the background). Please place these in the lower right corner of the images themselves. Please do not write on or near the bars in the image but define the size in the respective figure legend.

9) Please also note our reference format:
http://www.embopress.org/page/journal/14693178/authorguide#referencesformat

10) We updated our journal's competing interests policy in January 2022 and request authors to consider both actual and perceived competing interests. Please review the policy https://www.embopress.org/competing-interests and update your competing interests if necessary. Please name this section 'Disclosure and Competing Interests Statement' and put it after the Acknowledgements section.

11) We now use CRediT to specify the contributions of each author in the journal submission system. CRediT replaces the author contribution section. Please use the free text box to provide more detailed descriptions and do not provide your final manuscript text file with an author contributions section. See also our guide to authors:
https://www.embopress.org/page/journal/14693178/authorguide#authorshipguidelines

12) Please add 5 keywords to manuscript (below the abstract) and order the manuscript sections like this, using these names: Title page - Abstract - Keywords - Introduction - Results - Discussion - Materials and Methods - Data availability section - Acknowledgements - Disclosure and Competing Interests Statement - References - Figure legends - Expanded View Figure legends

We would also encourage you to use 'Structured Methods', our new Materials and Methods format. According to this format, the Materials and Methods section should include a Reagents and Tools Table (listing key reagents, experimental models, software and relevant equipment and including their sources and relevant identifiers) followed by a Methods and Protocols section in which we encourage the authors to describe their methods using a step-by-step protocol format with bullet points, to facilitate the adoption of the methodologies across labs. More information on how to adhere to this format as well as downloadable templates (.doc or .xls) for the Reagents and Tools Table can be found in our author guidelines (section 'Structured Methods'):

https://www.embopress.org/page/journal/14693178/authorguide#manuscriptpreparation

Moreover, I would need from you:
- a short, two-sentence summary of the manuscript (not more than 35 words).
- two to four short bullet points highlighting the key findings of your study (two lines each).
- a schematic summary figure as separate file that provides a sketch of the major findings (not a data image) in jpeg or tiff format (with the exact width of 550 pixels and a height of not more than 400 pixels) that can be used as a visual synopsis on our website.

Finally, please note that all corresponding authors are required to supply an ORCID ID for their name upon submission of a revised manuscript. Please do that for co-corresponding author John Marioni. Please find instructions on how to link the ORCID ID to the account in our manuscript tracking system in our Author guidelines:
http://www.embopress.org/page/journal/14693178/authorguide#authorshipguidelines

I look forward to seeing a revised version of your manuscript when it is ready. Please let me know if you have questions or comments regarding the revision.

Yours sincerely,

Achim Breiling
Senior Editor
EMBO Reports

Referee #1:

The revised manuscript is greatly improved. The data are all clear and convincing, and the experiments are all performed rigorously and with appropriate controls.

One minor suggestion is to label the effector T cells in Figure 4E as 'RAB27A/B-deficient' for clarity.

Referee #2:

My main comments were revolving around the way CTL infiltration was monitored and its relevance to an infected tissue or a tumor microenvironment. Those issues have been adequately addressed in the revised manuscript.

Referee #3:

The authors have neither addressed the concern that the very high E:T ratio may conceal the effect of actinomycin D on target cell killing. Likewise, convincing evidence that the aggregation assay represents CTL infiltration into a tumor-like environment remains to be provided. Without ruling out these points, the central line of reasoning that de-novo RNA synthesis and translation are not required for cytolysis but relevant for mediating CTL infiltration into tumors remains poorly supported by the data.

Fig. 3A lacks demonstration whether the effects of ActD on cytolysis remains unchanged at lower E:T ratios. Because an E:T ratio of 10:1 is excessive, the assay may not be sensitive enough to detect effector function under more realistic conditions. Is unperturbed killing in the presence of ActD retained at E:T ratio of 4:1 or 2:1 (which still are high)? This may uncover a role for translation in cytolysis when CTL densities are not supra-optimal.

Fig. 3D shows the confrontation of unlabeled CTL with fluorescent target cells, with the outcome of multi-cell aggregation and formation of an optically opaque, multicell sphere. The authors interpret this process as infiltration of CTL into a target cell layer, but no data are provided support this claim. Alternative processes underlying this process may be increased cell attachment followed by firm aggregation, which is actually is expected to limit migration due to firm cell-cell contacts. As also stated by reviewer 2, more direct evidence for infiltration or chemotaxis should be performed, e.g., by live-cell microscopy with single-cell resolution in a spheroid confrontation assay.

Minor

The data in Fig. 3 continue to lack statistical analysis.

Response to reviewer 1:

One minor suggestion is to label the effector T cells in Figure 4E as 'RAB27A/B-deficient' for clarity.

Many thanks to the reviewer for noticing this. The label has been added.

Response to reviewer 3:

We think that perhaps reviewer 3 is still confused about some important points in the manuscript. This reviewer thinks that we have not responded adequately to their earlier criticism from the first round of review where they stated:

"The effect of ActD on target cell killing is not well represented, across independent experiments and lack statistical analysis. Which portion of killing depends on de-novo synthesis of granule content, for example after 1h versus 4 h of inhibition of translation?"

We would like to politely point out that the reviewer seems to be confused about the use of Actinomycin D, which is a transcriptional inhibitor, not as they suggest a translational inhibitor.

This confusion seems to have surfaced again in the current comments where the reviewer states that our "central line of reasoning that de-novo RNA synthesis and translation are not required for cytolysis". This is certainly not our central line of reasoning. The current manuscript asks only what the role of early transcription is in CTL killing. *(We have previously addressed the role of translation in serial killing, showing that it has a role in re-filling of granules -see Lisci et al, Science 2021).*

The reviewer now asks an additional question which continues to confuse the role of Actinomycin D that they seem to think inhibits translation:
"Fig. 3A lacks demonstration whether the effects of ActD on cytolysis remains unchanged at lower E:T ratios. Because an E:T ratio of 10:1 is excessive, the assay may not be sensitive enough to detect effector function under more realistic conditions. Is unperturbed killing in the presence of ActD retained at E:T ratio of 4:1 or 2:1 (which still are high)? This may uncover a role for translation in cytolysis when CTL densities are not supra-optimal."

Regarding the E:T ratio, we have added back data that was included in our original submission using a lactate dehydrogenase release assay to measure target cell death 4h after CTLs were mixed with target cells at CTL:target ratios of 10:1, 5:1, 2.5:1 and 1.25:1 (new FigEV3D). These data show that although there is a trend toward reduction in target cell lysis with ActD treatment at all ratios (as was seen in the 10:1 live imaging assay), the effect is mild and doesn't substantially change with different CTL:target ratios. These data are supported by the degranulation assay we included (FigEV3B), which shows robust CTL degranulation in the presence of ActD, and our proteomics experiments (Fig2), which shows substantial expression of granule components was maintained after ActD treatment.

In this revision, we have also added data on how ActD treatment impacts target cell number (FigEV3C), demonstrating that ActD-induced death and/or inhibition of proliferation has negligible effects on target cell detection through 5h after treatment. Many hours after exposure, ActD does detrimentally impact target cell detection, and likely the health of CTLs as well, and thus "serial killing" assays that use low CTL:target ratios and later readouts are not feasible. To emphasize that our results and conclusions are based on these first several hours after actinomycin D treatment, we have added the word "several" to the abstract for clarity.

The next query from R3 is:
"Fig. 3D shows the confrontation of unlabeled CTL with fluorescent target cells, with the outcome of multi-cell aggregation and formation of an optically opaque, multicell sphere. The authors interpret this process as infiltration of CTL into a target cell layer, but no data are provided support this claim," asking for "more direct evidence for infiltration or chemotaxis"
Figure 3D is the quantitation of the primary data provided in Figure 3B and 3C and Source Data Fig3B Movies 2-3 and Fig3C Movies 1-2. These movies, images and quantitation clearly show the CTLs moving into the bolus of red target cells (or not when treated with ActD) and quantitate the changes in area. In addition, we provide a chemotaxis assay in Figure 4 that shows that "CTLs must engage in de novo transcription to initiate swarming behaviour" as stated in the text.

The reviewer's suggestion that "Alternative processes underlying this process may be increased cell attachment followed by firm aggregation, which is actually is expected to limit migration due to firm cell-cell contacts" is contradicted by our data that ActD treated CTLs migrate well on ICAM1 (Expanded View Fig4). Moreover, when 50% of the cells are treated with ActD in the co-culture assay (Figure 4E-F), this doesn't stop the other 50% from infiltrating and killing, so it's not making an impenetrable wall.

The reviewer's final minor comment also seems to arise from their missing data that is already included in the figure:
"Minor: The data in Fig. 3 continue to lack statistical analysis."

Both p values and ratios between conditions are provided in graphs in Figure 3. Figure 3A: Left panel is a representative example, middle panel is summarized over biological replicates with p-values and ratios, and right panel replots the summarized data to visually demonstrate the ratio effect sizes. Figures 3B-D: B-C are representative examples, while D summarizes measurements over biological replicates with p-values and ratios.

Dear Prof. Griffiths,

Thank you for the submission of your further revised manuscript to our editorial offices. I now went through your point-by-point response and the revised manuscript and consider the remining points of the referees as adequately addressed.

Before proceeding with formal acceptance, I have these further editorial requests I ask you to address:

- It seems something is missing from the title. I would suggest:
Cytotoxic T lymphocytes require transcription for infiltration but not for targeting cell lysis

- Please provide the abstract written in present tense.

- We plan to publish your manuscript in the Report format (as it has less than 25000 characters and 4 main figures). For a Scientific Report we require that results and discussion sections are combined in a single chapter called "Results & Discussion". Please do this for your manuscript. For more details, please refer to our guide to authors:
http://www.embopress.org/page/journal/14693178/authorguide#researcharticleguide

- Regarding data quantification and statistics, please make sure that the number "n" for how many independent experiments were performed, their nature (biological versus technical replicates), the bars and error bars (e.g. SEM, SD) and the test used to calculate p-values is indicated in the respective figure legends of all the figures. Please also check that all the p-values are explained in the legend, and that these fit to those shown in the figure. Please provide statistical testing where applicable. Please avoid the phrase 'independent experiment', but clearly state if these were biological or technical replicates. Please also indicate (e.g. with n.s.) if testing was performed, but the differences are not significant. In case n=2 please show the data as separate datapoints or bars without error bars and statistics. See also:
http://www.embopress.org/page/journal/14693178/authorguide#statisticalanalysis

If n<5, please show single datapoints for diagrams. Please update the legend for Fig. EV4C as indicated in your letter and make sure the final revised version of the figure is uploaded.

- Please make sure that all the funding information is also entered into the online submission system and that it is complete and similar to the one in the acknowledgement section of the manuscript text file. Presently, EMBL core funding is only mentioned in the acknowledgements.

- Tables EV1 and EV2 are datasets. Please upload these unzipped and renamed to Dataset EV1 and Dataset EV2 and as datasets. Please provide the legends as a separate sheet (TAB) in each Excel file. Finally, please change their callouts in the manuscript text file.

- Please make sure that all figure panels are called out separately and sequentially (main, and EV figures). Presently, There are no callouts for most of the movies. See also below.

- Each movie needs to be named Movie EV# and called out in the manuscript text file. Rach movie needs to be zipped with its corresponding legend (as text file) and uploaded as one folder.

- Please provide slightly thicker scale bars for panel EV4A without text in the image. There is also a second bar in these images, which text that can't be read. Please remove these or explain what these show in the legend. If these are important, they need to be shown larger so that the text is readable.

- Finally, please find attached a word file of the manuscript text (provided by our publisher) with changes we ask you to include in your final manuscript text. Please use the attached file as basis for further revisions and provide your final manuscript file with track changes, in order that we can see any modifications done.

Please also update the Data availability section, providing links to the datasets and codes and remove the referee usernames and passwords. Please make sure these are public latest upon publication of the paper.

I look forward to seeing the final revised version of your manuscript when it is ready. Please let me know if you have questions regarding the revision.

Please use this link to submit your revision: https://embor.msubmit.net/cgi-bin/main.plex

Best,

Achim Breiling

Senior Editor
EMBO Reports

The authors have addressed all minor editorial requests.

2nd Revision - Editorial Decision 25th Sep 2023

Prof. Gillian Griffiths
Cambridge Institute for Medical Research
Wellcome/MRC Building
Cambridge Biomedical Campus
Cambridge, Cambridgeshire CB2 0XY
United Kingdom

Dear Prof. Griffiths,

I am very pleased to accept your manuscript for publication in the next available issue of EMBO reports. Thank you for your contribution to our journal.

At the end of this email I include important information about how to proceed. Please ensure that you take the time to read the information and complete and return the necessary forms to allow us to publish your manuscript as quickly as possible.

As part of the EMBO publication's Transparent Editorial Process, EMBO reports publishes online a Review Process File to accompany accepted manuscripts. As you are aware, this File will be published in conjunction with your paper and will include the referee reports, your point-by-point response and all pertinent correspondence relating to the manuscript.

If you do NOT want this File to be published, please inform the editorial office within 2 days, if you have not done so already, otherwise the File will be published by default [contact: emboreports@embo.org]. If you do opt out, the Review Process File link will point to the following statement: "No Review Process File is available with this article, as the authors have chosen not to make the review process public in this case."

Thank you again for your contribution to EMBO reports and congratulations on a successful publication. Please consider us again in the future for your most exciting work.

Yours sincerely,

Achim Breiling
Senior Editor
EMBO Reports

****

THINGS TO DO NOW:

Please note that you will be contacted by Wiley Author Services to complete licensing and payment information. The required 'Page Charges Authorization Form' is available here: https://www.embopress.org/pb-assets/embo-site/er_apc.pdf - please download and complete the form and return to embopressproduction@wiley.com

EMBO Press participates in many Publish and Read agreements that allow authors to publish Open Access with reduced/no publication charges. Check your eligibility: https://authorservices.wiley.com/author-resources/Journal-Authors/open-access/affiliation-policies-payments/index.html

You will receive proofs by e-mail approximately 2-3 weeks after all relevant files have been sent to our Production Office; you should return your corrections within 2 days of receiving the proofs.

Please inform us if there is likely to be any difficulty in reaching you at the above address at that time. Failure to meet our deadlines may result in a delay of publication, or publication without your corrections.

All further communications concerning your paper should quote reference number EMBOR-2023-57653V3 and be addressed to emboreports@wiley.com.

Should you be planning a Press Release on your article, please get in contact with emboreports@wiley.com as early as possible, in order to coordinate publication and release dates.

EMBO Press Author Checklist

Corresponding Author Name: Gillian Griffiths	
Journal Submitted to: EMBO Reports	
Manuscript Number: 2023-57653-T	

USEFUL LINKS FOR COMPLETING THIS FORM
The EMBO Journal - Author Guidelines
EMBO Reports - Author Guidelines
Molecular Systems Biology - Author Guidelines
EMBO Molecular Medicine - Author Guidelines

Reporting Checklist for Life Science Articles (updated January
This checklist is adapted from Materials Design Analysis Reporting (MDAR) Checklist for Authors. MDAR establishes a minimum set of requirements in transparent reporting in the life sciences (see Statement of Task: 10.31222/osf.io/9sm4x). Please follow the journal's guidelines in preparing your
Please note that a copy of this checklist will be published alongside your article.

Abridged guidelines for figures
1. Data
The data shown in figures should satisfy the following conditions:
- the data were obtained and processed according to the field's best practice and are presented to reflect the results of the experiments in an accurate and unbiased manner.
- ideally, figure panels should include only measurements that are directly comparable to each other and obtained with the same assay.
- plots include clearly labeled error bars for independent experiments and sample sizes. Unless justified, error bars should not be shown for technical
- if n<5, the individual data points from each experiment should be plotted. Any statistical test employed should be justified.
- Source Data should be included to report the data underlying figures according to the guidelines set out in the authorship guidelines on Data

2. Captions
Each figure caption should contain the following information, for each panel where they are relevant:
- a specification of the experimental system investigated (eg cell line, species name).
- the assay(s) and method(s) used to carry out the reported observations and measurements.
- an explicit mention of the biological and chemical entity(ies) that are being measured.
- an explicit mention of the biological and chemical entity(ies) that are altered/varied/perturbed in a controlled manner.
- the exact sample size (n) for each experimental group/condition, given as a number, not a range;
- a description of the sample collection allowing the reader to understand whether the samples represent technical or biological replicates (including how many animals, litters, cultures, etc.).
- a statement of how many times the experiment shown was independently replicated in the laboratory.
- definitions of statistical methods and measures:
  - common tests, such as t-test (please specify whether paired vs. unpaired), simple χ2 tests, Wilcoxon and Mann-Whitney tests, can be unambiguously identified by name only, but more complex techniques should be described in the methods section;
  - are tests one-sided or two-sided?
  - are there adjustments for multiple comparisons?
  - exact statistical test results, e.g., P values = x but not P values < x;
  - definition of 'center values' as median or average;
  - definition of error bars as s.d. or s.e.m.

Please complete ALL of the questions below.
Select "Not Applicable" only when the requested information is not relevant for your study.

Materials

Newly Created Materials	Information included in the manuscript?	In which section is the information available? (Reagents and Tools Table, Materials and Methods, Figures, Data Availability Section)
New materials and reagents need to be available; do any restrictions apply?	Not Applicable	

Antibodies	Information included in the manuscript?	In which section is the information available? (Reagents and Tools Table, Materials and Methods, Figures, Data Availability Section)
For **antibodies** provide the following information:<br>- Commercial antibodies: RRID (if possible) or supplier name, catalogue number and or/clone number<br>- Non-commercial: RRID or citation	Yes	Materials and Methods: CTL culture and stimulation (p 13), flow cytometry (p 15), and degranulation (p 16) sections

DNA and RNA sequences	Information included in the manuscript?	In which section is the information available? (Reagents and Tools Table, Materials and Methods, Figures, Data Availability Section)
**Short novel DNA or RNA including primers, probes**: provide the sequences.	Not Applicable	

Cell materials	Information included in the manuscript?	In which section is the information available? (Reagents and Tools Table, Materials and Methods, Figures, Data Availability Section)
**Cell lines:** Provide species information, strain. Provide accession number in repository **OR** supplier name, catalog number, clone number, and/**OR** RRID.	Yes	Materials and Methods: Cell lines section (p 12)
**Primary cultures:** Provide species, strain, sex of origin, genetic modification status.	Yes	Materials and Methods: Mice (p 12), and CTL culture and stimulation sections (p 12)
Report if the cell lines were recently **authenticated** (e.g., by STR profiling) and tested for mycoplasma contamination.	Yes	Materials and Methods: Cell lines section (p 12)

Experimental animals	Information included in the manuscript?	In which section is the information available? (Reagents and Tools Table, Materials and Methods, Figures, Data Availability Section)
**Laboratory animals or Model organisms:** Provide species, strain, sex, age, genetic modification status. Provide accession number in repository **OR** supplier name, catalog number, clone number, **OR** RRID.	Yes	Materials and Methods: Mice (p 12), and CTL culture and sitmulation sections (p 12)
**Animal observed in or captured from the field:** Provide species, sex, and age where possible.	Not Applicable	
Please detail **housing and husbandry conditions**.	Yes	Materials and Methods: Mice section (p 12)

Plants and microbes	Information included in the manuscript?	In which section is the information available? (Reagents and Tools Table, Materials and Methods, Figures, Data Availability Section)
**Plants:** provide species and strain, ecotype and cultivar where relevant, unique accession number if available, and source (including location for collected wild specimens).	Not Applicable	
**Microbes:** provide species and strain, unique accession number if available, and source.	Not Applicable	

Human research participants	Information included in the manuscript?	In which section is the information available? (Reagents and Tools Table, Materials and Methods, Figures, Data Availability Section)
If collected and within the bounds of privacy constraints report on age, sex and gender or ethnicity for all study participants.	Not Applicable	

Core facilities	Information included in the manuscript?	In which section is the information available? (Reagents and Tools Table, Materials and Methods, Figures, Data Availability Section)
If your work benefited from core facilities, was their service mentioned in the acknowledgments section?	Yes	Acknowledgements (p 19)

Design

Study protocol	Information included in the manuscript?	In which section is the information available? (Reagents and Tools Table, Materials and Methods, Figures, Data Availability Section)

If study protocol has been **pre-registered, provide DOI in the manuscript**. For clinical trials, provide the trial registration number **OR** cite DOI.	Not Applicable	
Report the **clinical trial registration number** (at ClinicalTrials.gov or equivalent), where applicable.	Not Applicable	

Laboratory protocol	Information included in the manuscript?	In which section is the information available? (Reagents and Tools Table, Materials and Methods, Figures, Data Availability Section)
Provide DOI OR other citation details if **external detailed step-by-step protocols** are available.	Not Applicable	

Experimental study design and statistics	Information included in the manuscript?	In which section is the information available? (Reagents and Tools Table, Materials and Methods, Figures, Data Availability Section)
Include a statement about **sample size** estimate even if no statistical methods were used.	Yes	Materials and methods: CTL culture and stimulation section (p 13)
Were any steps taken to minimize the effects of subjective bias when allocating animals/samples to treatment (e.g. **randomization procedure**)? If yes, have they been described?	Yes	Materials and Methods: RNA-sequencing section (p 13)
Include a statement about **blinding** even if no blinding was done.	Yes	Materials and Methods: CTL culture and stimulation (p 13), and RNA-sequencing (p 13) sections
Describe **inclusion/exclusion criteria** if samples or animals were excluded from the analysis. Were the criteria pre-established?<br><br>If sample or data points were omitted from analysis, report if this was due to attrition or intentional exclusion and provide justification.	Yes	Materials and Methods: Live imaging cytolytic activity assay (p 17), Confocal live imaging (p 18), and Chemotaxis sections (p 19)
For every figure, are **statistical tests** justified as appropriate? Do the data meet the assumptions of the tests (e.g., normal distribution)? Describe any methods used to assess it. Is there an estimate of variation within each group of data? Is the variance similar between the groups that are being statistically compared?	Yes	Materials and Methods: RNA-sequencing analysis (p 14), Proteomics analysis (p 14-15), and Additional statistical analysis (p 19) sections

Sample definition and in-laboratory replication	Information included in the manuscript?	In which section is the information available? (Reagents and Tools Table, Materials and Methods, Figures, Data Availability Section)
In the figure legends: state number of times the experiment was **replicated** in laboratory.	Yes	Figure legends (p25-30)
In the figure legends: define whether data describe **technical or biological replicates**.	Yes	Figure legends (p25-30)

Ethics

Ethics	Information included in the manuscript?	In which section is the information available? (Reagents and Tools Table, Materials and Methods, Figures, Data Availability Section)
Studies involving **human participants**: State details of **authority granting ethics approval** (IRB or equivalent committee(s), provide reference number for approval.	Not Applicable	
Studies involving **human participants**: Include a statement confirming that **informed consent** was obtained from all subjects and that the experiments conformed to the principles set out in the WMA Declaration of Helsinki and the Department of Health and Human Services Belmont Report.	Not Applicable	
Studies involving **human participants:** For publication of **patient photos**, include a statement confirming that consent to publish was obtained.	Not Applicable	
Studies involving experimental **animals**: State details of **authority granting ethics approval** (IRB or equivalent committee(s), provide reference number for approval. Include a statement of compliance with ethical regulations.	Yes	Materials and methods: Mice section (p 12)
Studies involving **specimen and field samples:** State if relevant **permits** obtained, provide details of authority approving study; if none were required, explain why.	Not Applicable	

Dual Use Research of Concern (DURC)	Information included in the manuscript?	In which section is the information available? (Reagents and Tools Table, Materials and Methods, Figures, Data Availability Section)
Could your study fall under dual use research restrictions? Please check biosecurity documents and list of **select agents and toxins** (CDC): https://www.selectagents.gov/sat/list.htm	Not Applicable	
If you used a select agent, is the security level of the lab appropriate and reported in the manuscript?	Not Applicable	
If a study is subject to dual use research of concern regulations, is the name of the **authority granting approval and reference number** for the regulatory approval provided in the manuscript?	Not Applicable	

Reporting

The MDAR framework recommends adoption of discipline-specific guidelines, established and endorsed through community initiatives. Journals have their own policy about requiring specific guidelines and recommendations to complement MDAR.

Adherence to community standards	Information included in the manuscript?	In which section is the information available? (Reagents and Tools Table, Materials and Methods, Figures, Data Availability Section)
State if relevant guidelines or checklists (e.g., **ICMJE, MIBBI, ARRIVE, PRISMA**) have been followed or provided.	Yes	Materials and Methods: Mice section (p 12; relevant details are included throughout the manuscript)
For **tumor marker prognostic studies,** we recommend that you follow the **REMARK** reporting guidelines (see link list at top right). See author guidelines, under 'Reporting Guidelines'. Please confirm you have followed these guidelines.	Not Applicable	
For **phase II and III randomized controlled trials**, please refer to the **CONSORT** flow diagram (see link list at top right) and submit the CONSORT checklist (see link list at top right) with your submission. See author guidelines, under 'Reporting Guidelines'. Please confirm you have submitted this list.	Not Applicable	

Data Availability

Data availability	Information included in the manuscript?	In which section is the information available? (Reagents and Tools Table, Materials and Methods, Figures, Data Availability Section)
Have **primary datasets** been deposited according to the journal's guidelines (see 'Data Deposition' section) and the respective accession numbers provided in the Data Availability Section?	Yes	Data availability section (p 19)
Were **human clinical and genomic datasets** deposited in a public access-controlled repository in accordance to ethical obligations to the patients and to the applicable consent agreement?	Not Applicable	
Are **computational models** that are central and integral to a study available without restrictions in a machine-readable form? Were the relevant accession numbers or links provided?	Yes	Data availability section (p 19)
If publicly available data were reused, provide the respective **data citations in the reference list**.	Not Applicable	
